# Rewriting Polymer Fate via Chemomechanical Coupling

**DOI:** 10.1002/adma.202518567

**Published:** 2026-02-03

**Authors:** Jiahe Huang, Haohui Zhang, Jiehao Chen, Xuelin Sui, Febby Krisnadi, Dongjing He, Huajian Ji, Will R. Gutekunst, Michael D. Dickey, Yuhang Hu

**Affiliations:** ^1^ School of Chemical and Biomolecular Engineering Georgia Institute of Technology Atlanta Georgia USA; ^2^ George W Woodruff School of Mechanical Engineering Georgia Institute of Technology Atlanta Georgia USA; ^3^ School of Chemistry and Biochemistry Georgia Institute of Technology Atlanta Georgia USA; ^4^ Department of Chemical and Biomolecular Engineering North Carolina State University Raleigh North Carolina USA

**Keywords:** chemomechanics, dynamic polymers, growable soft electronics, transformable soft robots

## Abstract

How can synthetic polymers be endowed with the continuous, life‐like ability to grow, degrow, heal, and alter their chemical and physical properties after fabrication? This study addresses this question by coupling theory and experiment to create an open‐system “living” polymer platform that integrates mass transport, reversible polymerization, chain exchange, and evolving elasticity into a fully chemomechanically coupled network. Controlled transport, reaction, and stresses enable continuous growth and degrowth with microscale control enabled by light‐activated catalysts. Their chemical composition can be reprogrammed on demand, tuning modulus by up to two orders of magnitude to either stiffen or soften the material. These capabilities enable self‐growable electronics, transformative soft robots, and on‐site damage‐regenerating devices, establishing a foundation for sustainable, endlessly reprogrammable polymers.

## Graphical abstract

This work introduces a life‐like “living” polymer platform that can grow, degrow, and reprogram its properties after fabrication. By integrating mass transport, reversible polymerization, and controlled catalysis, the material achieves on‐demand changes in size, shape, and mechanical properties. Such dynamic behavior has enabled self‐growable electronics, transformable soft robots, and regenerating devices, paving the way for reprogrammable applications.

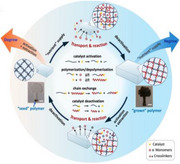

## 1 Introduction

Unlike living materials that constantly adjust their structure and properties to adapt to ever‐changing environments, traditional synthetic materials are typically static in both form and function. In recent years, reconfigurable materials, such as stimuli‐responsive hydrogels [1, 2], shape‐memory polymers [3, 4], magnetically responsive elastomers [5], and liquid crystal elastomers [6, 7], have emerged as promising alternatives. These materials offer shape reconfigurability and responsiveness, enabling new applications in robotics, biomedical devices, and wearable electronics. However, their reconfigurability remains limited, because their network backbones are fixed post‐fabrication, restricting them to switch between only a few predefined configurations upon stimulation. Overcoming this limitation calls for a shift in design philosophy—from incorporating a few switchable motifs to harnessing dynamic polymer chemistries that allow for post‐fabrication remodeling of both network structure and chemical composition. Dynamic polymer chemistries have traditionally been employed to design polymer architectures [8, 9, 10], enable reprocessing and recycling [11, 12, 13], and promote self‐healing [14, 15]. More recent work extends these concepts to post‐fabrication tuning of material [16, 17, 18, 19, 20, 21] and structural properties [22, 23, 24, 25, 26]. Despite exciting progress, this direction remains in its infancy, leaving vast opportunities to harness dynamic polymer chemistry for next‐generation repurposable, reusable, and recyclable materials. The key limitation is the absence of a platform that couples transport, reaction, and deformation into one coherent process. Unlike natural living materials, where these functions are inseparably intertwined, current synthetic systems treat them in isolation. Bridging this gap demands chemomechanically integrated polymers that unify nutrient transport, chemical remodeling, and mechanical transformation—an advance that has the potential to shift the field from incremental improvements toward truly life‐like, adaptive materials.

**FIGURE 1:**
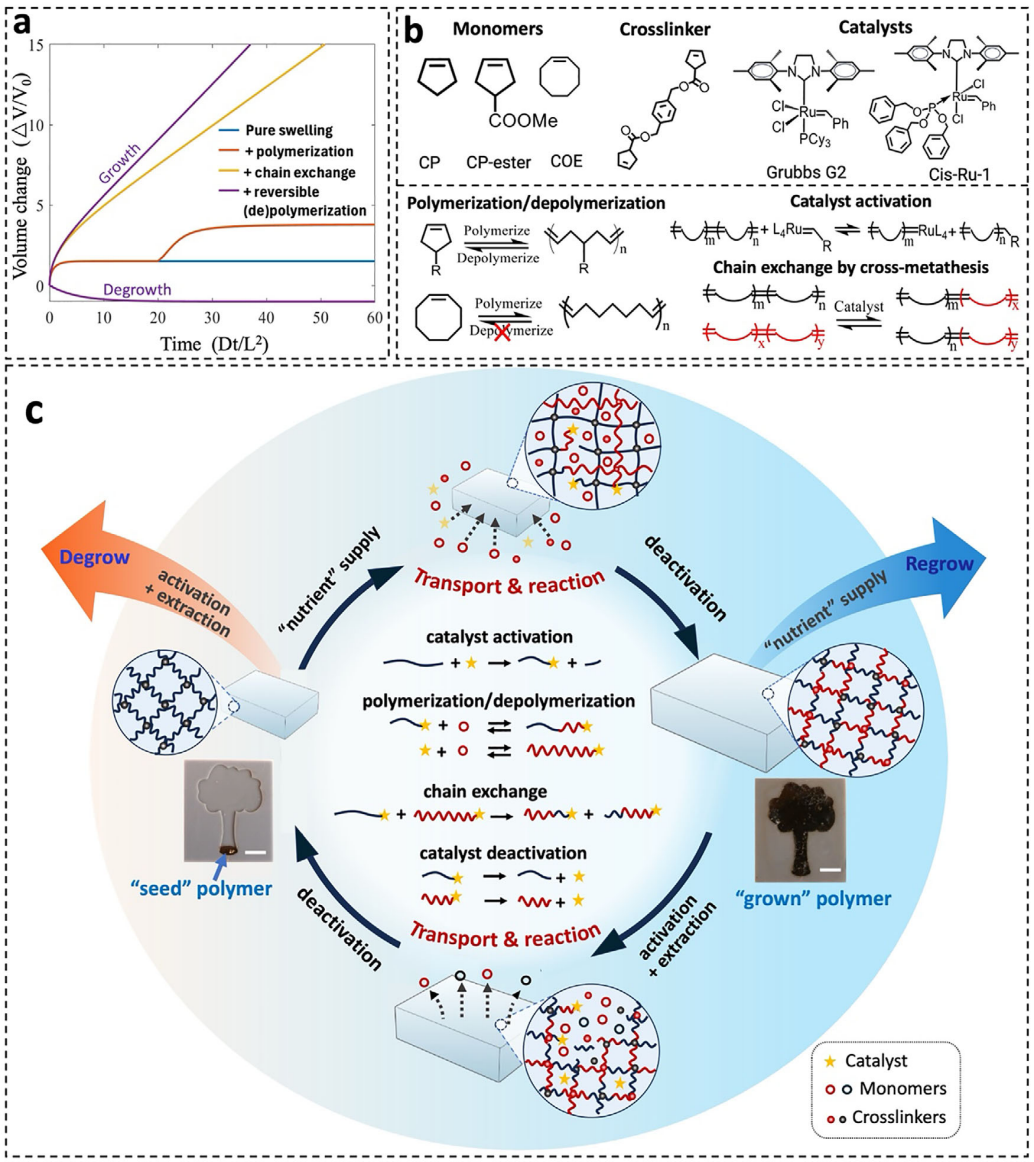
Design mechanism of the dynamic “living” polymer. (a) Computational illustration comparing the different growth behaviors and limitations of dynamic polymer systems governed by different reaction mechanisms. The time is normalized by diffusion time scale with *D* being the diffusivity and *L* the size of the sample. (b) Chemical structures of the monomers, crosslinker, and catalysts used in this work, along with the associated mechanisms for polymerization, depolymerization, catalyst activation, and chain exchange reactions. (c) Schematic representation of how concurrent processes—including catalyst activation/deactivation, polymerization/depolymerization, chain exchange, molecular transport, and mechanical deformation—collectively drive the macroscopic phenomena of growth, degrowth, and regrowth in the “living” polymer system. The pictures show a “living” polymer growing from a seed‐like structure into a tree‐like form within a confined environment, supported by a continuous nutrient supply. The scale bar is 1 cm. Details are in Supporting Information.

Here, we propose a different approach to dynamic polymer design by harnessing open systems and non‐equilibrium conditions, moving beyond traditional equilibrium‐based closed‐system strategies. By integrating concurrent reactions, mass transport, and mechanics through combined theory and experiment, we aim to develop a new class of dynamic “living” polymers capable of extensive post‐fabrication reprogramming in size, shape, and mechanical properties, thereby mimicking biological adaptability. To identify the minimal set of mechanisms required for continuously tunable polymers, we developed a statistical chain‐based model that incorporates chain remodeling reactions, mass transport, and evolving network elasticity (details in Supporting Information). When a crosslinked polymer network is exposed to an external solution with a higher chemical potential, it swells until it reaches its maximum extension, at which point swelling ceases — a behavior characteristic of typical hydrogels (blue line, Figure 1a). When the absorbed solution contains unreacted monomers and polymerization is initiated, monomer influx continues, driving further swelling (orange line, Figure 1a). However, as the new network forms and stiffens, further monomer absorption is constrained, resembling self‐strengthening double‐network hydrogels [19]. To overcome this mechanical limitation, chain exchange reactions can be introduced to alleviate the build‐up stress within the network, enabling additional monomer absorption and facilitating continuous polymer growth (yellow line, Figure 1a). However, if the kinetics of chain exchange lag significantly behind polymerization, stress accumulates in the original network and inhibits growth – consistent with the limited behaviors in previous attempts in realizing growing polymers [24]. To achieve reversible and tunable material transformations, reversible polymerization/depolymerization can be incorporated. These allow the material to not only grow but also undergo degrowth, regulated by external chemical potential: a higher potential promotes monomer uptake and growth, while a lower potential favors monomer expulsion and degrowth (purple lines, Figure 1a). Elasticity is also crucial—if the network is highly crosslinked, even a lower external chemical potential may be insufficient to drive the monomer transport due to prohibitive elastic energy. Therefore, achieving continuous and reversible tunability requires a delicate balance among reaction kinetics, transport rates, and evolving network elasticity.

Accordingly, the choice of chemistry to realize such “living” polymers requires careful considerations. The first challenge is balancing reactivity and stability: overly reactive chemistries make polymers too sensitive to environmental conditions, while excessive stability limits reprogrammability. Effective reversibility requires similar thermodynamic energy levels between monomers and polymers during polymerization, along with a chain exchange rate comparable to polymerization to relieve network stress and enable continuous growth. The polymer network must also maintain sufficient mechanical flexibility to facilitate osmotic‐driven monomer transport. For broad tunability, the chemistry must support diverse reaction rates, monomer types, and functional modifications. Prior work using cyclosiloxanes [18] partially demonstrated the concept of “living” polymers but suffered from slow reaction, poor spatiotemporal regulation, narrow mechanical tunability, and a single monomer type without functional modification. To fully realize chemomechanical coupling and the full potential of the “living” polymer concept in designing functional polymers, in this work, we adopt ring‐opening metathesis polymerization (ROMP), which offers a reversible monomer ⇌ polymer equilibrium governed by monomer structure, temperature, and concentration [27]. This equilibrium can be readily tuned by external conditions, while the shared cross‐metathesis mechanism enables polymerization, depolymerization, and chain exchange to be collectively regulated. Decades of catalyst development provide a wide range of tunable reactivities, enabling precise kinetic control [28, 29]. Additionally, ROMP offers a diverse monomer library with varying ring‐strain energies, facilitating tailored reaction kinetics and material properties. Monomer modifications further enable fine‐tuning of critical properties such as vapor pressure, viscosity, diffusivity, reactivity, crystallinity, and glass transition temperature. The reaction mechanism and representative monomers, crosslinkers, and catalysts are illustrated in Figure 1b. While ROMP serves as a proof of concept, these design principles apply broadly to other dynamic covalent chemistries, establishing a foundation for continuously tunable polymer systems.

The expected polymer behavior and working mechanisms are illustrated in Figure 1c. The starting polymer is synthesized via ROMP of monomers (e.g., CP‐ester) and a crosslinker, followed by catalyst deactivation and drying, resulting in a stable polymer. The material is then supplied with a “nutrient” solution containing monomer, crosslinker, and catalyst. As the nutrient is absorbed into the network, the catalyst interacts with the original polymer chains via olefin metathesis, anchoring onto the network and initiating polymerization of absorbed monomers onto the existing chains. Additionally, the catalyst and absorbed monomers can react to form new polymer chains. Through cross‐metathesis, newly formed and original polymer chains exchange segments, relieving swelling‐induced stress, enabling continuous uptake of nutrients. Upon catalyst deactivation and drying, a stable “grown” polymer is obtained. By adjusting crosslink density or monomer type in the nutrient solution, the grown polymer can exhibit different mechanical properties from the starting polymer. At this stage, the material can regrow if nutrient solutions are reintroduced. Conversely, degrowth can occur when the catalyst is reintroduced, and depolymerized small molecules are extracted from the network through evaporation. This process can also be applied to the starting polymer to further degrow it. These concurrent mechanisms enable controlled and continuous modulation of polymer size, shape and physical and chemical properties post‐fabrication.

## 2 Results and Discussion

### 2.1 Bulk Growth, Degrowth, and Degradation

**FIGURE 2:**
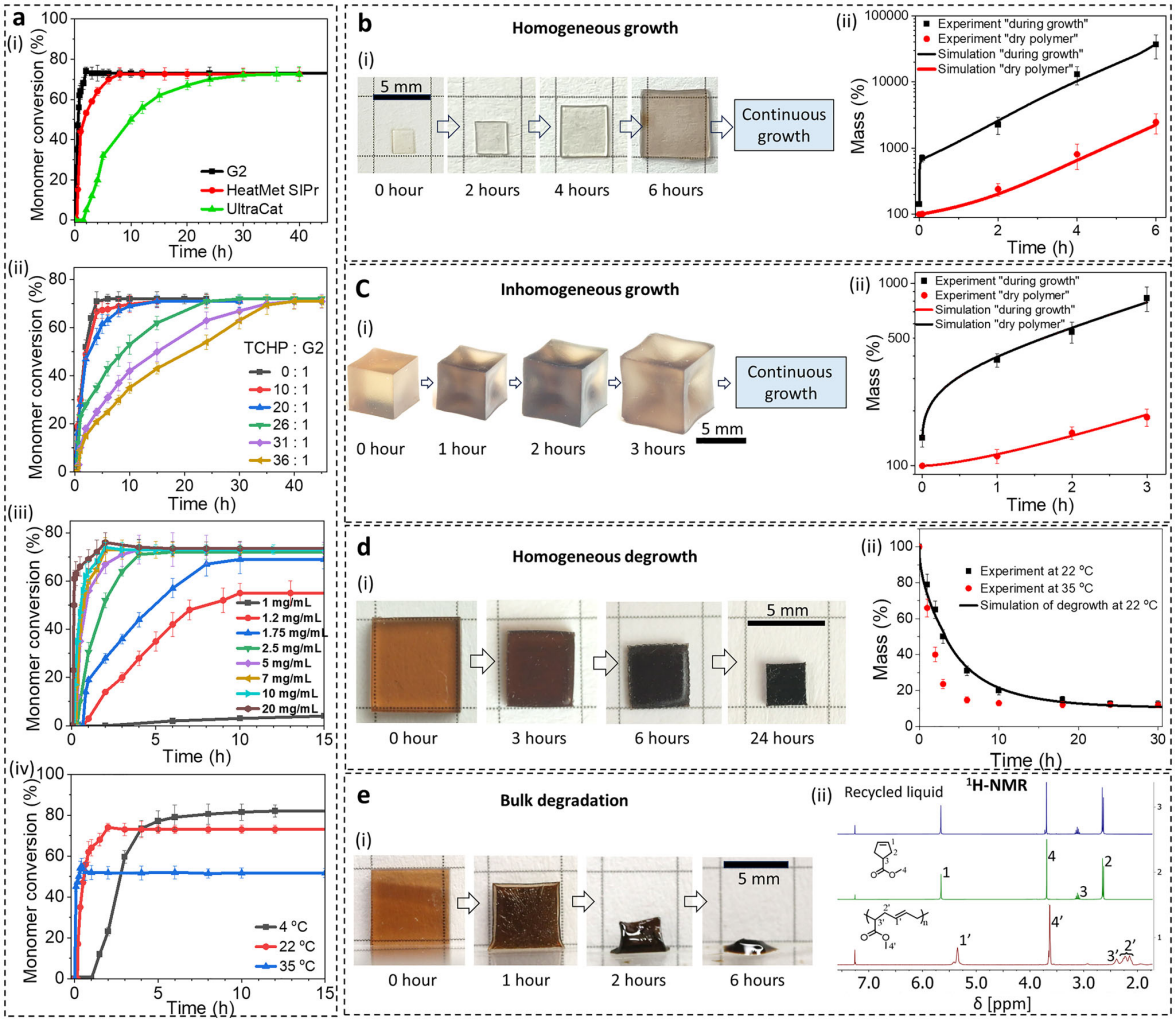
Bulk growth, degrowth, and degradation. (a) ROMP kinetics of CP‐ester (i) under different catalysts (10 mg/mL catalyst, 22°C), (ii) with varying TCHP to G2 molar ratios (2.5 mg/mL G2, 22°C), (iii) at different G2 concentrations (22°C) and (iv) at different reaction temperatures (10 mg/mL G2). (b) Homogeneous growth. (i) Images of 4 identical poly(CP‐ester) thin film samples (0.12 mm thick films with 2 vol.% crosslinker) shown in their initial state and after 2, 4, and 6 h of growth in a nutrient solution containing 98 vol.% CP‐ester, 2 vol.% crosslinker, 2.5 mg/mL G2, and 31:1 of TCHP:G2. All images were taken on the dry polymers after the samples were removed from the nutrient solution, deactivated, washed, and dried. (ii) Experimental and simulation results showing the mass change of the polymer films during growth in the nutrient solution and in their dry state. (c) Inhomogeneous growth. (i) Images of four poly (CP‐ester) cubes (2% crosslinker), shown in their initial state, and after 1, 2, and 3 h of growth in a nutrient solution containing 98 vol.% CP‐ester, 2 vol.% crosslinker, 7 mg/mL G2. All images show the polymers in their dry state. (ii) Experimental and simulation results of the mass change of the polymer cubes during growth in the nutrient solution and after drying. (d) Degrowth with shape retention. (i) Images of a 1 mm thick poly(CP‐ester) film (4 vol.% crosslinker and 10 mg/mL G2) before and after degrowth in an oven at 22°C for 3, 6 and 24 h. (ii) Experimental and simulation results of mass change in the film during degrowth at 22°C, along with experimental results of the same poly(CP‐ester) films degrading at 35°C. (e) Degradation upon collapse. (i) Images of a 1 mm thick poly(CP‐ester) film (4 vol.% crosslinker and 10 mg/mL G2), in the initial state and after degradation at 35°C for various durations. (ii) ^1^H‐NMR spectra of the liquid recovered from the degrading sample at 35°C, compared with spectra of pure CP‐ester and linear poly(CP‐ester). Error bars in all plots represent the standard deviation from 5 measurements.

Controlling growth requires balancing two competing timescales: transport of nutrient solutions and the chemical reaction. To tune reaction kinetics, we explored various ruthenium catalysts—Grubbs G2 (G2), HeatMet SIPr, UltraCat, and a mixture of G2 and tricyclohexyl phosphine (TCHP)— catalyst concentrations, TCHP:G2 ratios, and reaction temperature, and characterized monomer conversion rate via proton nuclear magnetic resonance (^1^H‐NMR) (Figure 2a; Figure S2). When transport is faster than reaction, the material undergoes homogeneous growth. To demonstrate this, four identical thin films of poly(CP‐ester) (thickness = 0.12 mm) were immersed in a nutrient solution with slow reaction kinetics (2.5 mg/mL G2, TCHP:G2 = 31:1 in molar ratio) for 5 min, 2, 4, and 6 h, followed by catalyst deactivation through washing in ethyl vinyl ether, and drying. The resulting images show uniform growth (Figure 2b), attributed to the rapid transport process (swelling equilibrium in ≈5 min, Figure S3a) compared to the slower reaction (≈80 min for 10% monomer conversion, Figure 2a(ii)). Conversely, when reaction outpaces transport, inhomogeneous growth occurs. This was demonstrated using poly(CP‐ester) cubes (5.3 × 5.3 × 5.3 mm^3^) grown in a monomer solution with fast reaction kinetics (7 mg/mL G2). Without the active catalyst, the cubes took ≈30 h to reach swelling equilibrium (Figure S3b), while polymerization achieved 10% monomer conversion in just 18 min (Figure 2a(iii)). As monomers diffuse inward, they are rapidly consumed, leading to faster polymerization at the outer layers. Corners, exposed on three surfaces, experience the fastest growth, resulting in a structure with protruded corners and an inhomogeneous shape (Figure 2c; Figure S4). Regardless of whether growth is homogeneous or inhomogeneous, continuous monomer supply and active catalyst sustain polymer expansion. Notably, neither diffusion time nor reaction time alone fully defines growth kinetics—it is a coupled process influenced by transport, reaction, polymer and monomer concentrations, network elasticity, and evolving material properties. To better understand this complexity, we developed a finite element model in COMSOL 6.0 based on the theory detailed in the Supporting Information. Simulations of thin films and polymer cubes closely matched experimental mass changes in both the growing and dried states (Figure 2b(ii),(ii)).

Leveraging the reversible nature of ROMP of cyclopentene, we demonstrate the degrowth of the “living” polymers. The CP‐ester monomer, a liquid at room temperature, evaporates in an open environment. When a 1‐mm thick poly(CP‐ester) film with active catalyst was exposed to air, monomer evaporation disrupted the monomer‐polymer equilibrium, driving depolymerization. As shown in Figure 2d, at 22°C with gradual monomer evaporation, the polymer exhibits uniform size reduction. To validate the degrowth mechanism, we simulated the process using the finite element code. It shows strong agreement with experimental data (Figure 2d(ii)). At 35°C, increased catalyst activity and accelerated monomer diffusion prevented effective network reorganization, causing structural collapse under gravity, referred to as “degradation” (Figure 2e). The evaporated chemicals were collected and analyzed using ^1^H‐NMR and GC/MS (Figure S6). It shows that only CP‐ester monomer was extracted through this process (Figure 2e(ii); Figure S6b), while crosslinkers and oligomers remained in the network because of the specific chemical potential condition of the environment.

### 2.2 Photo‐Controlled Local Growth and Degradation

**FIGURE 3:**
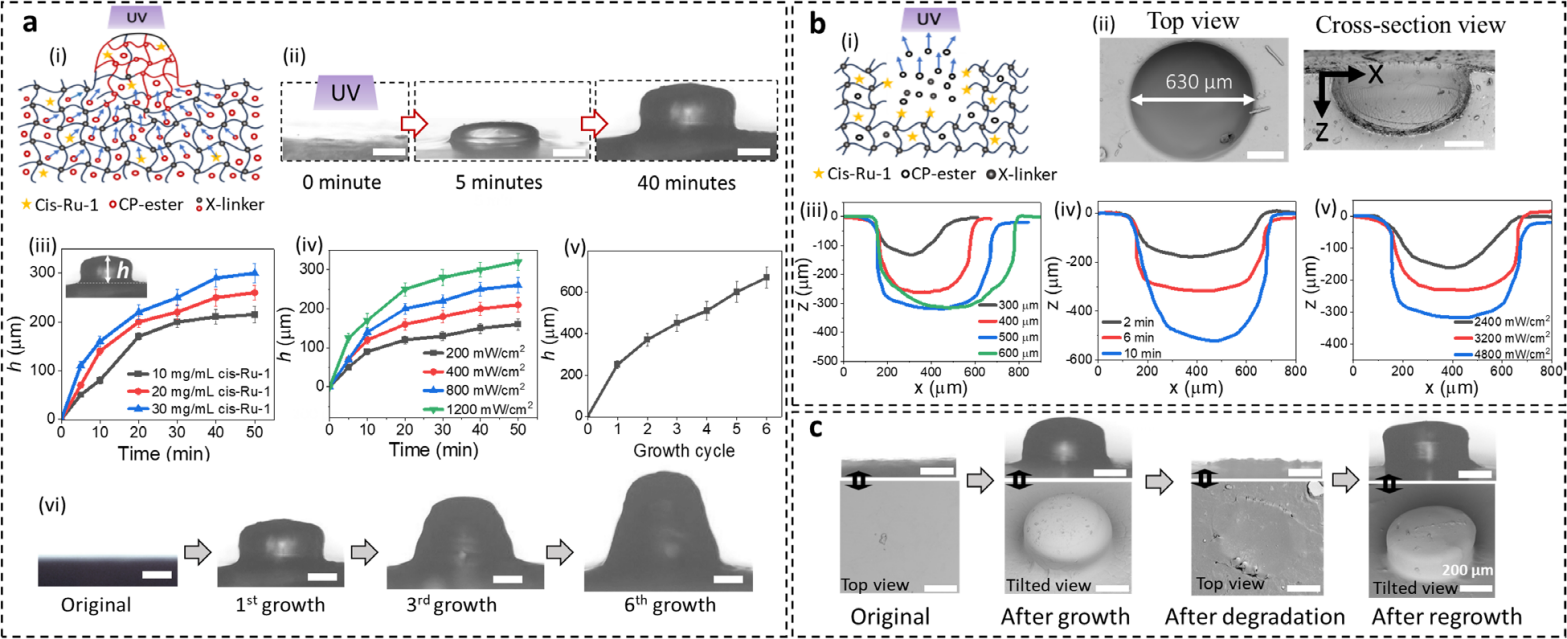
Light‐induced local growth and degradation. (a) Local growth. (i) Schematic illustration of the mechanism of light‐induced local growth in the dynamic “living” polymer. (ii) Optical microscopic images showing local growth in a poly(CP‐ester) sample swollen with a nutrient solution (97 vol.% CP‐ester, 3 vol.% crosslinker, 10 mg/ml cis‐Ru‐1), following ultraviolet (UV) light exposure (405 nm, 800 mW/cm^2^, 500 µm diameter) for 5 and 40 min. The height of the grown micro‐pillar (measured from the center of the pillar) plotted against irradiation time at (iii) different cis‐Ru‐1 concentrations, and (iv) different light intensities. (v) Micro‐pillar height across successive growth cycles. For each cycle, a fresh nutrient solution was supplied, followed by 40 min UV exposure (vi) Microscopic images of the grown micro‐pillar after the first, third^,^ and sixth growth cycles. (b) Local degradation. (i) Schematic illustration of the mechanism of light‐induced local degradation in the dynamic “living” polymer. (ii) Scanning electron microscope (SEM) images on the top and cross‐sectional views of a micro‐hole formed on a poly(CP‐ester) sample containing active catalyst (without nutrient solutions), exposed to UV light (405 nm, 600 µm diameter, 4800 mW/cm^2^, 6 min). (iii) Cross‐sectional profiles of micro‐holes formed under UV exposure (405 nm) with varying (iii) light dot diameters (4800 mW/cm^2^, 6 min), (iv) irradiation time (4800 mW/cm^2^, 500 µm diameter), and (v) light intensity (500 µm diameter, 6 min). (c) Repetitive local growth, degradation, and regrowth. A poly(CP‐ester) sample was made and noted as “original.” After the sample was swollen in a nutrient solution (97 vol.% CP‐ester, 3 vol.% crosslinker, 10 mg/ml cis‐Ru‐1) and exposed to UV light (405 nm, 500 µm light dot diameter, 800 mW/cm^2^, 40 min), a micro‐pillar was grown on the surface. Next, the sample was reinfused with 2% cis‐Ru‐1 and exposed to UV light (405 nm, 500 µm light dot diameter, 4800 mW/cm^2^, 6 min), leading to the degradation of the previously grown pillar. Finally, after another nutrient solution infusion and UV exposure (405 nm, 500 µm light dot diameter, 800 mW/cm^2^, 40 min), a new micro‐pillar regrew at the original location. All SEM images were taken on the dry polymer after it was deactivated, washed, and dried. Scale bars in all images represent 200 µm. Error bars denote standard deviation from 5 measurements.

To achieve precise spatial control, we explored photo‐triggered growth and degrowth/degradation using various photo‐initiated catalysts (Figure S8). One set of results is shown in Figure 3a. A poly(CP‐ester) sample was swollen in nutrient solution containing CP‐ester, crosslinker, and cis‐Ru‐1, then irradiated on a surface spot (diameter 500 µm) under 405 nm light. Light activation of cis‐Ru‐1 induced polymerization in the irradiated region, consuming local monomers and creating a chemical potential gradient that drove monomer diffusion from surrounding non‐irradiated areas to sustain localized growth (Figure 3a(i)). To quantify net growth, the catalyst was deactivated, and the sample was dried before all characterizations. Growth height increased with irradiation time but slowed as the monomer diffusion distance became longer (Figure 3a(ii‐iv)). Higher cis‐Ru‐1 concentrations accelerated polymerization, enhancing the chemical potential gradient and promoting faster diffusion and greater growth heights. Similarly, increased light intensity boosted catalyst activity, further enhancing growth. Additional effects of parameters such as irradiation area and polymer composition are detailed in Figures S10–S12. To overcome transport limitations on local growth height, we implemented multiple localized growth cycles. Each cycle involved supplying additional monomer solution and irradiating with 405 nm light for 40 min (Figure 3a(v, vi); Figure S14). Results demonstrated continuous localized growth to 6 cycles without capping observed.

Light‐activated catalysts not only enable local growth but also trigger localized depolymerization depending on the nutrient condition. Starting with a dry poly(CP‐ester) sample, we first swelled it with a cis‐Ru‐1/dichloromethane (DCM) solution without monomers. After DCM evaporation, the dry films contained cis‐Ru‐1. Local 405 nm light irradiation then activated cis‐Ru‐1, triggering depolymerization of poly(CP‐ester) while the generated monomers evaporated, further accelerating the process. This concurrent activation, depolymerization, and evaporation led to localized degradation (Figure 3b(i)). As shown in Figure 3b(ii), a circular light dot (405 nm, 4800 mW/cm^2^, 600 µm diameter) produced a micro‐hole with well‐defined dimensions; the hole diameter (≈630 µm) was only 5% larger than the illuminated region, demonstrating good resolution. Smaller light dot diameters produced correspondingly smaller holes (Figure 3b(iii)). Diameters below 200 µm showed no significant degradation after 6 min due to limited light power at that scale of our laser source. Hole depths increased with longer irradiation times while widths remained largely unchanged (Figure 3b(iv)). Similarly, higher light intensity and increased cis‐Ru‐1 concentration led to greater hole depths without affecting the surface width (Figure 3b(v); Figure S16). Importantly, even at the highest light intensity, the sample temperature only reached ≈27°C (Figure S17), much lower than in typical laser ablation, preventing significant chemical alterations and allowing subsequent regrowth. Furthermore, the catalyst cis‐Ru‐1 cannot be thermally activated even at 80°C (Figure S18), which enhances the precision of photo‐degradation by eliminating concerns regarding thermal activation of cis‐Ru‐1 and unintended degradation in non‐irradiated regions. To demonstrate reversible transformation, we grew a micro‐pillar on a poly(CP‐ester) sample using 405 nm light. After drying and re‐infusing with only cis‐Ru‐1 without monomers, the pillar was degraded under 405 nm light (Figure 3c). Reinfusing the sample with monomer solution followed by light irradiation allowed a new micro‐pillar to be grown, confirming the system's ability to support repeated growth and degradation cycles.

### 2.3 Modulating Mechanical Properties by Growth and Degrowth

**FIGURE 4:**
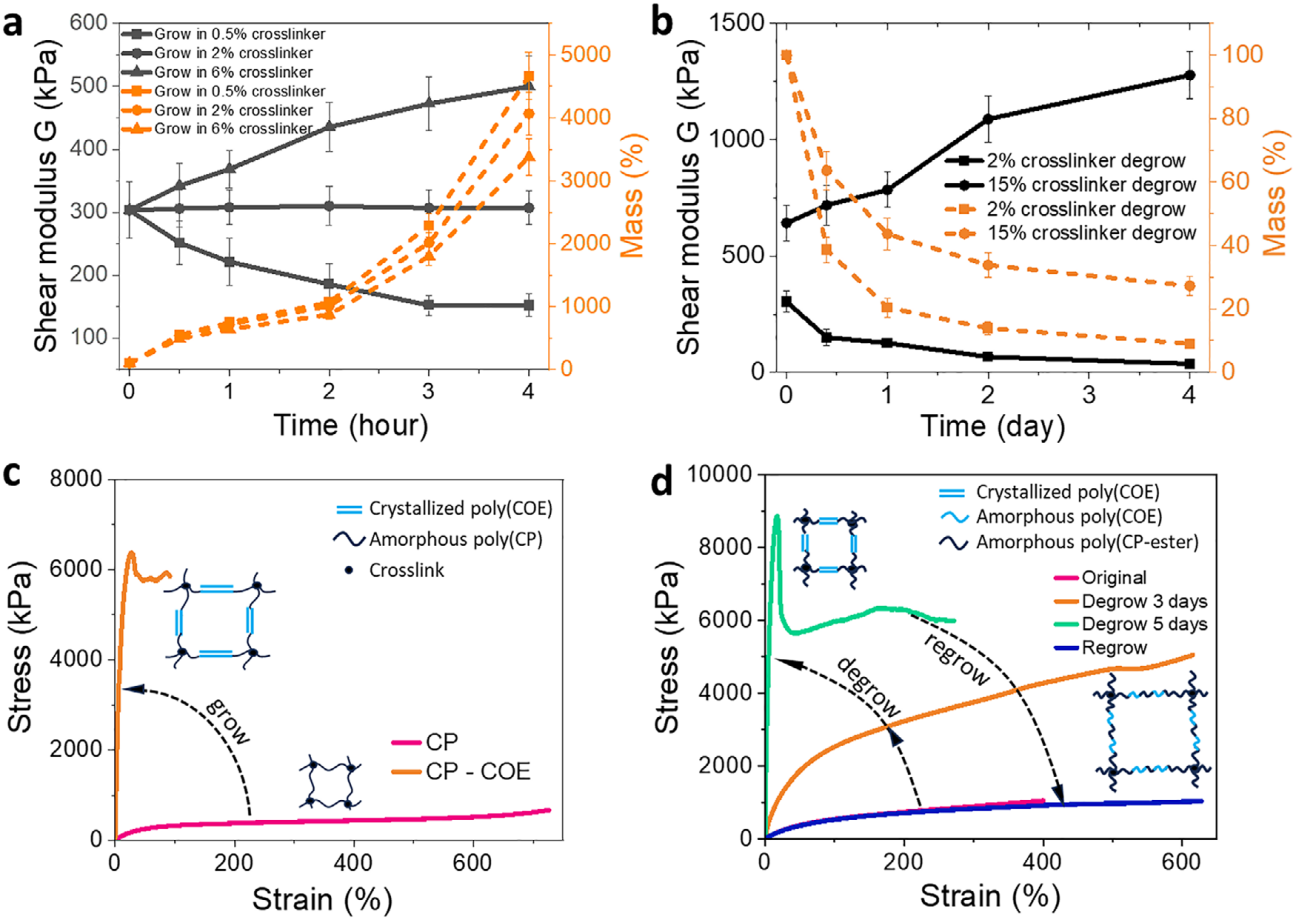
Reprogramming post‐fabrication mechanical properties through growth and degrowth.(a) Shear modulus G, extracted from the tensile testing using the Mooney‐Rivlin model, and mass change of the poly(CP‐ester) with 2 vol.% crosslinker at various growth stages in nutrient solutions containing 0.5, 2, and 6 vol.% crosslinkers. The nutrient solution also contains 2.5 mg/mL G2 and TCHP:G2 ratio of 18:1. (b) Shear modulus G and mass change during the degrowth process of poly(CP‐ester) synthesized with 2 vol.% crosslinker and 15 vol.% crosslinker, measured at different time points. (c) Tensile stress‐strain behaviors of a poly(CP) sample with 1 vol.% crosslinker (denoted as “CP”), compared to its behavior after growing in a nutrient solution containing 99 vol.% COE, 1 vol.% crosslinker and 1.2 mg/mL G2 for 30 min (denoted as “CP—COE”). The growth amount of the sample is ≈1700% in mass. (d) Tensile stress‐strain behaviors of a sample composed of 30 vol.% COE, 68 vol.% CP‐ester, and 2 vol.% crosslinker (denoted as “Original”) compared to the same sample after undergoing degrowth for 3 and 5 days and compared to the same sample after regrowth in a nutrient solution containing 98 vol.% CP‐ester and 2 vol.% crosslinker (denoted as “Regrow”) for 90 min. All tensile testing samples were dry polymers that had been deactivated, washed, and dried before testing. Error bars represent the standard deviation from 5 measurements.

The “living” polymer enables post‐fabrication tuning of mechanical properties. First, the modulus of the polymer can be modulated through the growth process by varying the crosslinker‐to‐monomer ratio in the nutrient solution. Five dry poly(CP‐ester) films (0.3 mm thick), all with an initial crosslinker density of 2%, were immersed in nutrient solutions with CP‐ester, G2 catalyst, and crosslinker concentrations of 0.5%, 2%, and 6%, respectively. After growth, the films were deactivated, washed, dried, and tested via uniaxial tensile testing (Figure S19a), with shear modulus G calculated using the Mooney‐Rivlin model (Figure 4a; Figure S19b)), wherein the Mooney‐Rivlin model fits well with experimental data up to 400% strain. As shown, grown in a 0.5% crosslinker solution, the “living” polymer is softened as its crosslinker density decreased through the growth process, grown in a 2% crosslinker solution, the polymer maintains its modulus, while growth in a 6% solution, the polymer is stiffened as crosslinker density increased. Second, the mechanical properties of the “living” polymer can also be tuned via degrowth. Poly(CP‐ester) thin films (1‐mm thickness) with active G2 catalyst were placed in an argon‐flow container (Figure S7a) for monomer evaporation. After degrowth, the samples were deactivated, washed, dried, and tested (Figure S20a). The shear modulus (Figure 4b; Figure S20b) revealed that lower crosslinker samples softened, while those with ≥6% crosslinker stiffened over time during degrowth. This effect arises from two competing mechanisms. While monomer evaporation increases crosslinker density, stiffening the network, the rising catalyst concentration enhances depolymerization and chain exchange, which can reduce stiffness. In low‐crosslinker samples, depolymerization dominates, leading to softening, while in highly crosslinked samples, increased crosslinker concentration prevails, resulting in stiffening. Thus, both growth‐ and degrowth‐induced mechanical changes can be tailored.

The diverse monomer options in ROMP chemistry enable more significant mechanical property tuning through growth and degrowth than through crosslinker density regulation. Here, we introduce cis‐cyclooctene (COE), which crystallizes after polymerization under certain conditions [30]. We use poly(cyclopentene) (poly(CP)) as the starting polymer due to its high swelling ratio (≈1800%) in COE, compared to 4.5% for poly(CP‐ester). As shown in Figure 4c, the shear modulus of poly(CP) (99% CP, 1% crosslinker) increased by two orders of magnitude, from 0.36 to 21.7 MPa, after growth in a monomer solution containing 99% COE and 1% crosslinker. This stiffening is attributed to poly(COE) crystallization after growth, confirmed by differential scanning calorimetry (DSC) (Figure S21b,c). Degrowth‐induced mechanical property changes can also be significant. Starting with a copolymer of CP‐ester and COE (COE:CP‐ester:crosslinker = 30:68:2), its shear modulus increased two orders of magnitude from 0.34 to 42.7 MPa after 5 days of degrowth (Figure 4d). During degrowth, poly(CP‐ester) depolymerizes and evaporates, while poly(COE) remains, leading to an increased poly(COE) fraction that promotes further crystallization (Figure S22b,c) and higher stiffness. A broader range of degrowth‐induced modulus changes can be achieved by adjusting the COE‐to‐CP‐ester ratio (Figure S22). Furthermore, after 5 days of degrowth, the stiff polymer can regrow in CP‐ester monomer solution to become soft again (Figure 4d). The dramatic changes in mechanical properties over multiple cycles are shown in Figure S23. A key to regrowth is melting the poly(COE) crystalline at 60°C, allowing swelling in a monomer solution (98% CP‐ester, 2% crosslinker, catalyst), which polymerizes and undergoes chain exchange with poly(COE), restoring softness (details in Experimental Section and Supporting Information). All the mechanical characterizations are on the stable polymers after catalyst deactivation and drying.

### 2.4 Engineering Applications

**FIGURE 5:**
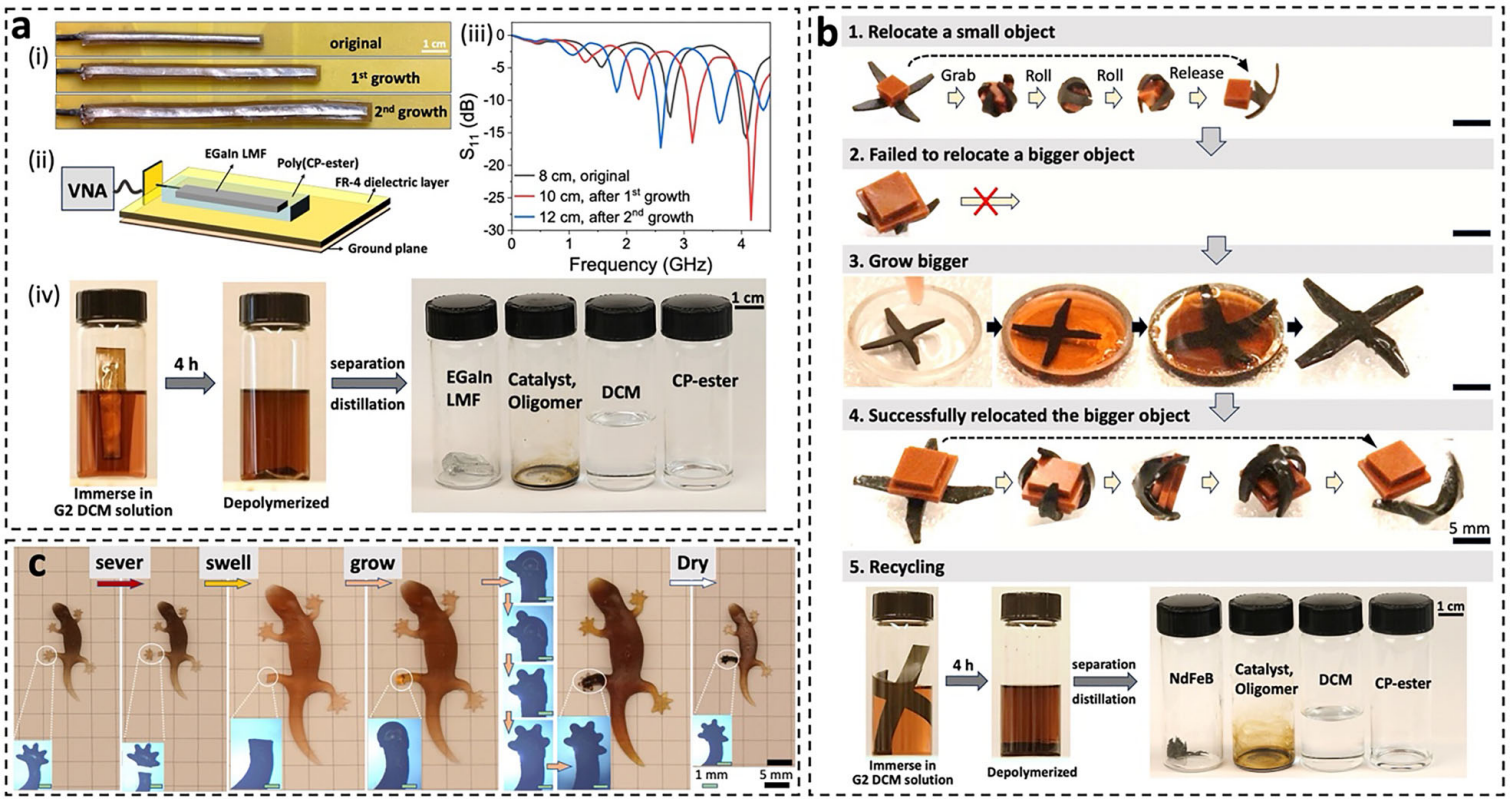
Exemplary applications of the dynamic “living” polymer. (a) Growable flexible antenna. (i) Images showing the antenna in its initial state and after the first and second rounds of growth. (ii) Schematic illustration of the setup used for radio frequency measurement of the antenna. (iii) S_11_ plotted against frequency, comparing the antenna's performance in its initial state and after each growth phase. (iv) Recycling process of the antenna, which involves depolymerization in a DCM and G2 solution, followed by distillation to recover CP‐ester and the EGaIn LMF. (b) Transformative soft robot. Illustration of a magnetic soft robot that undergoes growth to successfully relocate a larger object. The recycling process involves depolymerization in a DCM and G2 solution, followed by distillation to recover CP‐ester and NdFeB. (Video S1) (c) “Gecko” limb regeneration. A series of images depicting the regeneration of a gecko‐shaped poly(CP‐ester) structure. The sequence includes: molding the polymer into a gecko shape, cutting a limb, swelling it with a monomer solution, and sequentially growing the “foot sole” and “toes” under local light exposure. This is followed by deactivating G2, washing, and drying to complete the “gecko limb regeneration” process.

The “living” polymer developed in this study can integrate seamlessly with other engineering materials to create devices with continuously tunable properties, enabling repurposing, reprogramming, repair, and recycling. We first demonstrate this with a reconfigurable monopole antenna whose resonance frequency is tuned via polymer growth. The antenna was constructed by embedding eutectic gallium indium (EGaIn) liquid metal foam (LMF) [31] into a poly(CP‐ester) channel (Figure S25). Radio frequency testing was conducted using a vector network analyzer (VNA) connected via a 50 Ω coaxial cable (Figure 5a(ii)). Initially, the 8 cm long antenna exhibited its first resonant peak at 1.6 GHz (Figure 5a(iii)). To reprogram its frequency, the antenna was immersed in a nutrient solution (CP‐ester, 2% crosslinker, G2) and grown twice, each time extending by 2 cm in length (Figure 5a(i)). This shifted the first‐peak resonance to 1.3 GHz and 1.06 GHz, respectively (Figure 5a(iii)), thereby providing a wireless route to communicate the state of growth. The LMF deformed while maintaining conductivity during growth (Figure S26). However, grown further, the LMF discontinuity occurred. Besides tunable frequency, the antenna is also recyclable: immersion in DCM and catalyst solution depolymerizes the polymer back to monomers, enabling recovery and reuse of both the liquid metal foam and CP‐ester (Figure 5a(iv); Figure S27).

The dynamic polymers not only interface with other engineering materials easily but also enable composite fabrication. As a demonstration, we incorporated magnetic particles into the polymer to create a soft robot capable of size transformation. The robot, made of poly(CP‐ester) embedded with neodymium–iron–boron (NdFeB) microparticles, was magnetized with a programmed dipole distribution using a pulse magnetizer (≈1.5 Tesla, Figure S28). As shown in Figure 5b and Video S1, the robot was guided by a magnet (Figure S28c) to grab and relocate a small object. However, its limited size restricted it from handling larger objects. After growing in a nutrient solution of the same crosslink density as the original polymer, the robot expanded. Different from gel swelling that significantly softened the gel, this “living” polymer can maintain its mechanical integrity and be able to successfully handle a larger object after the transformation. Additionally, the robot is also recyclable: exposure to DCM and catalyst solution depolymerizes the polymer back to monomers, enabling recovery and reuse of both the polymer and magnetic particles.

Healing is a highly desirable property in engineering devices to reduce costly maintenance and repair. Beyond fusion‐type healing, this “living” polymer enables light‐driven regeneration of damaged parts. Using localized growth, we mimicked the gecko limb regeneration function. A poly(CP‐ester) sample molded into a “gecko” shape, and a limb of the “gecko” was cut. The gecko with a damaged hindlimb was then supplied with a nutrient solution and irradiated with 405 nm light locally. Light‐induced growth first regenerated the “foot sole” and then sequentially rebuilt the “toes” with localized light irradiation (Figure 5c). This example demonstrates fine‐controlled, post‐fabrication shape regeneration, highlighting the polymer's potential for free‐form manufacturing.

## 3 Conclusion

Through an open‐system nonequilibrium dynamic polymer design, this work demonstrates the feasibility of creating synthetic polymer with continuous post‐fabrication reprogrammability in size, shape and mechanical properties. Guided by a physics‐based theory and leveraging the versatile characteristics of reversible ROMP chemistry, a wide range of finely tuned engineering controls was achieved. By modulating environmental conditions, the polymer can be directed to grow or degrowth. Homogeneous and spatially inhomogeneous growth were realized by tuning the relative timescales of concurrent mass transport and chemical reactions. Similarly, by adjusting the elasticity, transport rate and reaction kinetics, the polymer can exhibit either uniform degrowth or degradation. The integration of a photocatalyst enabled localized growth and degradation, allowing for freeform, spatially resolved post‐fabrication structural modifications. Mechanical properties can be modulated by up to two orders of magnitude – either stiffened or softened—through growth and degrowth processes. These “living” polymers demonstrate broad potential for practical applications, as exemplified by a growable magnetic soft robot, reconfigurable monopole antenna, and “gecko limb regeneration.” We envision that such “living” polymers can reduce routine maintenance and be continuously reprogrammed for changing application requirements without requiring energy‐intensive and costly depolymerization and re‐synthesis. When needed, they can still be broken down into monomers for recycling. These capabilities can significantly extend the service life of polymers, reduce dependence on petroleum‐based feedstocks, and offer substantial economic and environmental benefits.

## 4 Experimental Section

### 4.1 Materials

All chemicals were used as received unless otherwise noted. UltraCat (95%), Grubbs catalyst M801, Grubbs catalyst M220 (cis‐Caz‐1), CDCl_3_ (99.5%), were purchased from Millipore Sigma. Ethyl vinyl ether (EVE, 99%), pyridine (99.5%), α,α′‐Dibromo‐p‐xylene (97%), Grubbs G2 (G2), Benzil (99%), 2‐isopropylthioxanthone(ITX, 98%), ethyl‐4‐dimethylamino benzoate (EDAB), cis‐cyclooctene (COE, 95%), HeatMet SIPr, dichloromethane (DCM), potassium carbonate, sylgard 184, and pentane were purchased from Fisher Scientific. Cyclopent‐3‐ene‐1‐carboxylate (CP‐ester, 97%), and cyclopent‐3‐ene‐1‐carboxylic acid (97%), were purchased from Chem Scene. The crosslinker 1,4‐phenylenebis(methylene) bis(cyclopent‐3‐ene‐1‐carboxylate) [20] and cis‐Ru‐1 [32] were synthesized according to literature procedures (details provided below). Neodymium‐iron‐boron (NdFeB) microparticles (5 µm) were purchased from Magnequench, Singapore.

### 4.2 Gas Chromatography‐Mass Spectrometry (GC/MS)

GC/MS analysis was performed using an Agilent system consisting of an 8860 gas chromatograph coupled with a 5977‐mass selective detector (MSD). The system was equipped with a capillary column, and electron impact (EI) ionization was used for mass spectrometric detection.

### 4.3 Differential Scanning Calorimetry (DSC)

DSC measurements were performed using a TA Instruments Discovery 250 differential scanning calorimeter. Samples were heated from −50°C to 150°C at a rate of 10°C/minute.

### 4.4 Tensile Testing

Tensile tests were conducted using an Instron universal tensile tester model 5944 with a 1 kN load cell. Experiments were carried out at room temperature with a strain rate of 100% strain per minute.

### 4.5 Scanning Electron Microscopy (SEM)

SEM imaging was carried out using a Phenom XL scanning electron microscope. Polymer samples were fully dried and sputter‐coated with gold prior to imaging. SEM was performed at an accelerating voltage of 5 kV.

### 4.6 Thermal imaging

Sample temperatures during light‐induced growth and degradation were recorded using a FLIR A6751 thermal camera equipped with a 25 mm lens (1–5 µm, f/2.5).

### 4.7 Micro‐Computed Tomography (Micro‐CT) Scan

Surface profiling of the polymer cubes was conducted using a Scanco µCT50 imaging system (SCANCO Medical, Brüttisellen, Switzerland). Samples were placed in a cylindrical holder (34 mm diameter) and fixed on the carousel sample stage. Scanning was performed using an X‐ray energy of 45 kV and a current of 133 µA, with a 0.1 mm aluminum filter. The integration time was set to 300 milliseconds, and the voxel size was 48.6 µm. Reconstructed 3‐D images were generated using the DICOM viewer software provided by the manufacturer.

### 4.8 Polymerization Kinetics Measurement

Monomer CP‐ester and catalyst were mixed and allowed to react at a specified temperature. After a predetermined reaction time, approximately 20 mg of the mixture was transferred to a nuclear magnetic resonance (NMR) tube, followed by the addition of 170 µL of CDCl_3_ and 30 µL of ethyl vinyl ether (EVE) to quench the catalyst. The mixture was vortexed to ensure thorough mixing, after which an additional 650 µL of CDCl_3_ was added. ^1^H‐NMR spectra were recorded on a 400 MHz Varian Mercury spectrometer. Chemical shifts (δ) are reported in ppm relative to the residual solvent peaks (CDCl_3_ at 7.26 ppm or CD_2_Cl_2_ at 5.12 ppm). Monomer conversion was calculated using the following equation:

The monomer conversion = Integral of H (5.27–5.45 ppm) / [(Integral of H (5.27–5.45 ppm) + Integral of H (5.62 – 5.7 ppm)] × 100%.

### 4.9 CP‐Ester Polymerization Kinetics Catalyzed with Photo‐Catalysts

A glass vial containing 300 µL of a mixture of CP‐ester and a selected catalyst (cis‐Caz‐1, cis‐Caz‐1 with Benzil, ITX, EDAB, or cis‐Ru‐1) was placed in a liquid bath consisting of 65% glycerol and 35% water. The bath sat atop a custom‐built 405 nm LED light array composed of 25 LEDs (3.5 V, 700 mA, 120° 1414 (3535 Metric), Digikey). The temperature of the liquid bath was maintained at approximately 10°C using a circulating chiller while not in reaction, which resulted in the internal temperature of the CP‐ester mixture reaching ≈22°C during light exposure (405 nm, 200 mW/cm^2^). Light intensity was regulated using a DDS signal generator. After irradiation for a predetermined duration, 20 mg of the reaction mixture was transferred to an NMR tube. Subsequently, 30 µL of EVE and 170 µL of CDCl_3_ were added, and the mixture was vortexed for several minutes. Finally, an additional 650 µL of CDCl_3_ was added before performing the ^1^H‐NMR analysis using a 400 MHz Varian Mercury spectrometer.

### 4.10 Growth of Thin Films for Homogeneous Growth

A thin film of poly(CP‐ester) containing 2 vol.% crosslinker was prepared by polymerizing a mixture of 98 vol.% CP‐ester, 2 vol.% crosslinker, 2.5 mg/mL Grubbs G2 catalyst, and 25.6 mg/mL tricyclohexylphosphine (TCHP) (TCHP : G2 = 31 : 1 in molar ratio). The polymerization was conducted in a mold formed by two glass plates separated by a 0.12 mm thick rubber spacer. After 40 h of reaction, the resulting film was removed and cut into square sheets (0.12 mm × 2.2 mm × 2.2 mm). Four of these sheets were immersed in a fresh monomer solution of identical composition (98 vol.% CP‐ester, 2 vol.% crosslinker, 2.5 mg/mL G2, and 25.6 mg/mL TCHP) to allow simultaneous monomer swelling and polymer growth. To prevent premature polymerization of the bulk monomer solution and entrapment of the sheets, the monomer solution was refreshed approximately every ≈80 min. At predetermined time points (5 min, 2, 4, and 6 h), one sheet was removed from the growth solution and transferred to a deactivation and washing solution consisting of 90% DCM and 10% EVE. Samples were put on a glass plate and partly covered with a petri dish to slowly evaporate the solvent for 2 h, followed by removing the petri dish and sitting in a fume hood to dry for 22 h in air. Dry films were imaged using a Canon EOS 80D digital camera and weighed using an analytical balance. Each experiment was repeated five times to determine the average mass change during the growth process. Growth experiments with varying catalyst compositions followed the same procedure, with adjusted polymerization and monomer refreshing times. Specifically, films polymerized with 2.5 mg/mL G2 and 29.8 mg/mL TCHP (TCHP : G2 = 36 : 1 in molar ratio) were cured for 45 h, and their monomer solutions were refreshed every 2.5 h. For films polymerized with 2.5 mg/mL G2 and 21.5 mg/mL TCHP (TCHP : G2 = 26 : 1 in molar ratio), the initial film polymerization time was 26 h, and monomer solutions were refreshed every 40 min.

### 4.11 Growth of Polymer Cubes

Polymer cubes were fabricated by molding a mixture of 98 vol.% CP‐ester, 2 vol.% crosslinker, and 7 mg/mL Grubbs G2 catalyst into polydimethylsiloxane (PDMS) molds (Sylgard 184, mixed at an 8:1 ratio of base to curing agent). The polymerization was allowed to proceed for 3 h, after which the cubes were demolded and used immediately for growth experiments. Three polymer cubes were immersed in a monomer solution identical in composition to that used for cube formation (98 vol.% CP‐ester, 2 vol.% crosslinker, 7 mg/mL G2). To prevent excessive viscosity that could hinder cube removal, the monomer solution was refreshed approximately every 16 min. At predetermined time points (1, 2, and 3 h), one cube was removed and immediately transferred into a quenching solution composed of 70 vol.% ethanol, 20 vol.% DCM, and 10 vol.% EVE to deactivate the catalyst and remove any unreacted monomer. Samples were then put onto a glass plate and covered with a petri dish to slowly dry for 48 h (solvents getting out from the gap between the petri dish and the glass plate). Dried cubes were imaged using a Canon EOS 80D digital camera, weighed using an analytical balance, and analyzed for surface topography using micro‐CT with a Scanco µCT50 system. Each growth condition was repeated five times to determine the average mass change during the growth process.

### 4.12 Degrowth and Degradation of Polymer Films

To investigate the effect of temperature on polymer degrowth, a mixture of 96 vol.% CP‐ester, 4 vol.% crosslinker, and 10 mg/mL Grubbs G2 catalyst was polymerized for 3 h to form a film. The resulting film was then cut into square sheets (≈1 mm × 5 mm × 5 mm), which were placed on a sheet of tissue paper (Kimtech Kimwipes) inside a temperature‐controlled oven (Across International, Stable Temp‐25 FO‐19070) set to 22°C. Mass change during the degrowth process was monitored over time using an analytical balance. Each experiment was repeated five times to obtain average mass loss values. Degradation experiments followed a similar procedure, with the primary differences being that the samples were placed on glass slides and the oven temperature was increased to 35°C to accelerate degradation. To study the effects of catalyst concentration and crosslinker density on degrowth, additional experiments were performed under an argon (Ar) atmosphere to facilitate the removal of volatile monomers. In these experiments, samples were placed in a sealed container with continuous Ar flow. The inlet flow rate of Ar was maintained at approximately 36 L/hour, as measured using a manual bubble flowmeter.

### 4.13 Light‐Induced Localized Growth

A base polymer film was prepared by molding a mixture of 98 vol.% CP‐ester, 2 vol.% crosslinker, and 10 mg/mL Grubbs G2 catalyst, followed by polymerization for 2 h. The film was then deactivated, washed, and dried. A sample (4 mm × 4 mm × 1.5 mm) was cut from the film and swollen in a monomer solution (CP‐ester, Crosslinker, and cis‐Ru‐1) at 4°C for 10 h. After swelling, the sample was placed on the stage of a Zeiss upright AXIO Scope A1 microscope equipped with a 405 nm light source (Mightex Polygon 1000). Light was directed onto the sample via the objective lens to initiate localized polymer growth. Following irradiation, the sample was immediately transferred into a quenching solution of 90% DCM and 10% EVE to deactivate G2 and remove unreacted monomers, then put on a glass plate and partly covered with a petri dish to slowly evaporate the solvent for 2 h, followed by removing the petri dish and sitting in a fume hood to dry for 22 h. Surface‐grown micropillars were visualized under the Zeiss microscope. For samples grown under high‐intensity light (3200 mW/cm^2^), micropillars were bisected with a razor blade and further characterized using SEM.

For multi‐cycle growth experiments, the dry polymer film (4 mm × 4 mm × 1.5 mm, 98 vol.% CP‐ester, 2 vol.% crosslinker) was swollen in a nutrient solution containing 97 vol.% CP‐ester, 3 vol.% crosslinker, and 20 mg/mL cis‐Ru‐1 at 4°C for 10 h. The sample was then irradiated with 405 nm light (800 mW/cm^2^, 500 µm diameter circular spot) for 40 min. This constituted the “first‐cycle” growth. After irradiation, the sample was quenched, washed, and dried.

For “second‐cycle” growth, samples were not quenched after the first irradiation. Instead, they were placed into a 2 mL vial surrounded by nutrient solution, with the light‐irradiated surface not in direct contact with the liquid. After 20 min of vapor‐phase exposure, the sample was fully immersed in nutrient solution for an additional 40 min, then irradiated again under the same light conditions for 40 min. Following this step, the sample was quenched, washed, and dried.

“Multi‐cycle” growth was achieved by repeating the following cycle:

1. 20 min of vapor‐phase swelling (without immersing the micropillar),
2. 40 min of full immersion in the nutrient solution, and
3. 40 min of 405 nm light irradiation.

All samples were quenched, washed, and dried only after completing all desired growth cycles. All growth experiments were repeated 5 times.

### 4.14 Light‐Induced Local Degradation

Poly(CP‐ester) samples (2 vol.% crosslinker, 4 mm × 4 mm × 1.5 mm), previously deactivated, washed, and dried, were immersed in a solution of DCM and cis‐Ru‐1 for catalyst infusion. The cis‐Ru‐1 concentration in the DCM solution was determined based on the swelling ratio ≈1350% of DCM swelling into poly(CP‐ester) (equal to 10.15 µL DCM swelling into 1 mg of poly(CP‐ester)), and samples were incubated in the solution for 4 h inside a sealed vial. Afterward, the vial cap was removed to allow DCM evaporation over 3 h while leaving the swollen samples at the bottom of the vial. Samples were then removed and placed under vacuum for 1 h to fully eliminate residual DCM, resulting in poly(CP‐ester) infused with cis‐Ru‐1. The infused samples were irradiated with 405 nm light using a Zeiss upright AXIO Scope A1 microscope equipped with a Mightex Polygon 1000 light source. Light exposure was applied for predetermined durations to induce localized degradation. Following irradiation, samples were quenched in a solution of 90% DCM and 10% EVE to deactivate the catalyst, washed thoroughly, and dried slowly for 24 h. Dried samples were sputter‐coated with gold and imaged using SEM to characterize the resulting micro‐holes. For cross‐sectional analysis, the micropatterned regions were bisected at the center using a razor blade, followed by gold sputter‐coating and SEM imaging of the cross‐section profiles.

### 4.15 Tuning Mechanical Properties by Growth

To investigate mechanical property tuning via post‐fabrication growth process, five dry poly(CP‐ester) films (0.3 mm thick, 2  vol.% crosslinker) were immersed in a monomer solution composed of CP‐ester, crosslinker (0.5  vol.%, 2 vol.% or 6 vol.%), 2.5 mg/mL Grubbs G2 catalyst, and 15 mg/mL TCHP (TCHP:G2 = 18:1) at room temperature for growth. The monomer solution was replaced with freshly prepared ones after every ≈45 min. After a certain time (0.5, 1, 2, 3, and 4 h), one film was taken out of the monomer solution, placed between two glass slides inside a closed vial under an argon atmosphere, where it underwent polymerization for 24 h. Following polymerization, samples were immersed in a solution of 90% DCM and 10% EVE to quench the catalyst and extract unreacted monomers. The samples were then put on a glass plate and partly covered with a petri dish to slowly evaporate the solvent for 2 h, followed by removing the petri dish and sitting in a fume hood to dry for 22 h to yield the final grown materials.

Mechanical testing was performed using an Instron universal testing machine under uniaxial tension at a strain rate of 100% strain per minute. Tensile stress–strain curves were collected up to 400% strain and fitted using the Mooney–Rivlin model to extract the material constants C_1_ and C_2_. The shear modulus (G) was calculated using the equation: G  =  2  ×  (C1  +  C2)

For poly(CP) growth experiments, thin films (0.1 mm thick, 99 vol.% CP, 1 vol.% crosslinker) were swollen in a monomer solution containing 99 vol.% cis‐cyclooctene (COE), 1 vol.% crosslinker, and 1.2 mg/mL G2 at 4°C for 30 min. The swollen films were then clamped between two glass slides and polymerized for 24 h. Afterward, G2 was quenched in pure EVE, and the samples were washed and dried.

All experiments were repeated five times to obtain average values for mass change and mechanical property variation.

### 4.16 Tuning Mechanical Properties by Degrowth and Regrowth

1 mm thick polymer films with active G2 (10 mg/mL) after polymerization for 5 h were put on tissue paper inside a container with flowing argon (∼36 L/hour). After a certain time, a fraction of the sample was cut out and put in a solution with DCM and EVE (10 vol.%) to quench G2, followed by drying on the bench for 24 h. The dried samples were then stretched on an Instron to get tensile properties at 100% strain per minute and tested with DSC 250 to quantify the crystallinity. For degrowth and regrowth of 30% COE: The polymer film of 30% COE (30 vol.% COE, 68 vol.% CP‐ester, 2 vol.% Crosslinker, 10 mg/mL G2) after polymerization for 3 h was first put in the container with argon flow to degrow for 5 days at 22°C, followed by quenching catalyst in DCM and EVE and drying 24 h. For regrowth, the degrown sample was submerged in a monomer solution (98 vol.% CP‐ester, 2 vol.% crosslinker, 10 mg/mL G2) at 60°C for 6 min to disrupt crystalline domains, with the external solution being replaced with freshly prepared ones every 2 min. The sample was then swollen in a second monomer solution (98 vol.% CP‐ester, 2 vol.% crosslinker, 13 mg/mL G2) at 4°C for 90 min to ensure homogeneous monomer, crosslinker and catalyst uptake, replacing the solution every 30 min. After swelling, the sample was removed and allowed to react for 5 h at 22°C under Ar, then quenched in 90% DCM/10 vol.% EVE to deactivate G2 and bench‐dried for 24 h. For subsequent cycles, regrown samples containing G2 were not quenched with EVE and were instead directly subjected to the same degrowth procedure as the initial cycle. All mass‐change measurements for growth–degrowth cycles were performed on fully dried samples after quenching, washing, and drying.

### 4.17 Crystallinity Calculation

The crystallinity of a polymer due to the crystallization of poly(COE) was calculated as *X*_c_ = ΔH_m_ / ΔH^0^_m_ × 100%, wherein *X*_c_ is the crystallinity, ΔH_m_ is the melting enthalpy of the sample at temperatures above 22°C, and ΔH^0^_m_ is the melting enthalpy of 100% crystalline poly(COE) (230 J/g) [33].

### 4.18 Antenna Preparation, Measurement, and Recycling

A mixture of 98 vol.% CP‐ester, 2 vol.% crosslinker, and 10 mg/mL G2 was used to fabricate the outer polymeric structure of the antenna. Two parts were molded. As shown in Figure S25, one part is a 7.7 cm × 5 mm × 1 mm strip featuring an open channel on the top with the dimensions of 7 cm × 2 mm × 0.5 mm. Another part is a 7.7 cm × 5 mm × 0.5 mm strip. EGaIn LMF, prepared by stirring EGaIn in the air following the procedure in literature [31], was introduced into the channel, which was then sealed by aligning and bonding the two poly(CP‐ester) parts. After 2‐h reaction at room temperature, the two parts were fully polymerized and chemically merged together. The assembled antenna was then immersed in a solution of 90 vol.% DCM and 10 vol.% EVE to wash off unreacted monomers and quench the catalyst, followed by slow drying for 24 h. A sharp needle was then used to pierce through the antenna, establishing a connection between the LMF and a coaxial cable for measuring the 1‐port scattering parameter S_11_ using the vector network analyzer (Keysight E5063A), across a frequency range of 3.5 to 5 GHz. After testing, the microcrack generated by the sharp needle was repaired by adding a drop of a monomer solution (98 vol.% CP‐ester, 2 vol.% Crosslinker, 20 mg/mL G2) to the surface. The monomer solution gradually diffused into the crack and sealed the crack after 2 h of polymerization, sealed the damage. For antenna growth, the sample was immersed in a solution of 98 vol.% CP‐ester, 2 vol.% Crosslinker, and 10 mg/mL G2 at 4°C for 45 min, then transferred to a closed container to polymerize at 22°C for 4 h. The antenna was then washed in the 90% DCM and 10% EVE solution to quench G2, remove unreacted monomers, and dried. The dried antenna was used for the radio frequency test. The subsequent growth follows the same procedure, with swelling times adjusted to 60 min for the second growth and 70 min for the third growth. For recycling, the antenna was placed DCM containing 2 mg/mL G2 and depolymerized for 3 h. The EGaIn LMF was separated from the solution, which was then heated up to 50°C in a rotary evaporator connected to a ‐40°C coolant to sequentially recover the DCM (boiling point 39.6°C) and CP‐ester (boiling point 149.8°C).

### 4.19 Magnetic Robot Preparation, Magnetization, Actuation, and Recycling

A mixture of CP‐ester (78 vol. %), crosslinker (2 vol. %), G2 (10 mg/mL), and NdFeB (20 vol. %) was thoroughly mixed in a syringe until the solution started to become viscous. The mixture was then quickly dispensed onto a mold consisting of a glass plate and a 0.5 mm rubber spacer, covered with another glass plate and polymerized for 3 h. After curing, the resulting film was immersed in a solution of DCM (90%) and EVE (10%) to quench G2 and remove unreacted monomers, followed by drying for 12 h. The dried polymer film was cut into a cross shape with tilted cuts at the ends of the four “arms” (Figure S28a). The four “arms” were bent, and the sample was positioned at the center of an electromagnetic coil to undergo magnetization using a lab‐made impulse magnetizer (≈1.5 Tesla at the center). Once magnetized, the soft robot could be actuated using a permanent neodymium magnet placed underneath a table, enabling it to move, grab, and relocate small objects. The growth process of the robot is shown in Video S1. The monomer solution used for growth contained 8 mg/mL G2, 2 vol.% crosslinker, and 98 vol.% CP‐ester. The recycling process followed the same procedure as that used for the antenna: the robot was immersed in DCM containing 2 mg/mL G2 to depolymerize for 3 h. The NdFeB particles were then separated from the solution by a magnet. The solution was then heated to 50°C in a rotary evaporator connected to a −40°C coolant to sequentially recover DCM and CP‐ester.

### 4.20 “Gecko Limb Regeneration” Via Light‐Induced Polymer Growth

A gecko‐shaped polymer was fabricated by molding a mixture of 98 vol.% CP‐ester, 2 vol.% crosslinker, and 20 mg/mL Grubbs G2 catalyst into a PDMS mold. After polymerization for 1 h, the gecko was demolded and immersed in a quenching solution of 90% DCM and 10% EVE to deactivate the catalyst. The sample was then dried slowly for 24 h. To simulate limb damage, the gecko's hind limb was amputated using a razor blade. The injured gecko was then swollen in a nutrient solution containing 97 vol.% CP‐ester, 3 vol.% crosslinker, and 20 mg/mL cis‐Ru‐1 at 4°C for 8 h. After swelling, the gecko was positioned under a Zeiss upright AXIO Scope A1 microscope equipped with a 405 nm Mightex Polygon 1000 light source. A 405 nm light pattern, matching the cross‐sectional shape of the amputated limb, was projected onto the site to induce polymerization with concurrent nutrient transport and form the “foot sole.” Subsequently, circular light spots (approximately 600 µm in diameter) were sequentially applied to grow the “toes.” Both the sole and toe structures required multiple cycles of nutrient solution swelling and light irradiation (800 mW/cm^2^) to achieve the desired morphology. To prevent unintended polymerization from prolonged catalyst activity, the gecko was quenched, washed, and dried after approximately 20 h from the start of the swelling process. Upon completion of the multi‐step growth procedure, the regenerated gecko was immersed in a quenching solution of 90% DCM and 10% EVE, washed thoroughly, and dried, yielding a gecko with a “regenerated hindlimb.” This “toe” regeneration process involves nutrient swelling and light irradiation without erasing steps and is unaffected by humidity, as ROMP proceeds efficiently in water and our hydrophobic catalyst–monomer system is insensitive to ambient moisture. Although pH effects on ROMP are highly catalyst‐ and monomer‐dependent, literature indicates that lower pH can modestly increase monomer conversion for Grubbs catalysts [34]. In practical laboratory conditions, regeneration is robust at 20–26°C with normal ventilation; key factors include maintaining the nutrient at 4°C during swelling to avoid unintended reactions and preventing excessive airflow that can dry the limb. Because CP‐ester ROMP is temperature‐dependent, regeneration may fail above ≈35°C.

## Author Contributions

Y.H. and J.H. conceptualized the research idea. Y.H. supervised the overall project. J.H. led the material development, including organic synthesis, material preparation, characterization, post‐programming, and application demonstrations. Y.H. and H.Z. developed the theoretical framework. H.Z. did the simulation. J.C. designed and built the magnetizer. X.S. and W.G. assisted with the cis‐Ru‐1 photo catalyst synthesis. F.K. and M.D. contributed to the realization of the growable antenna. D.H. conducted micro‐CT experiments. H.J. carried out the FTIR and tensile testing on some samples and the seed‐to‐tree demonstration. Y.H. wrote the main manuscript. J.H. wrote the Experimental Section and supporting documents. All authors reviewed the manuscript and contributed to its editing.

## Funding

This work was supported by the Office of Naval Research Award No. N00014‐23‐1‐2754.

## Conflicts of Interest

The authors declare no conflicts of interest.

## Supporting information




**Supporting File 1**: adma72336‐sup‐0001‐SuppMat.pdf.

**Supporting File 2**: adma72336‐sup‐0002‐VideoS1.mp4.

## Data Availability

The data that support the findings of this study are available in the supplementary material of this article.
