# Supplementary material for: Rewriting Polymer Fate via Chemomechanical Coupling
Source: Adv Mater. 2026 Feb 3;38(24):e18567. doi: 10.1002/adma.202518567 (PMC13113212; doi:10.1002/adma.202518567)
Supplement: Supplementary file 1 — Supporting File 1: adma72336‐sup‐0001‐SuppMat.pdf. [file ADMA-38-e18567-s001.pdf]

Rewriting Polymer Fate via Chemomechanical Coupling

*Jiahe Huang, Haohui Zhang, Jiehao Chen, Xuelin Sui, Febby Krisnadi, Dongjing He, Huajian Ji, Will R. Gutekunst, Michael D. Dickey, and Yuhang Hu**

The PDF file includes:

Synthesis

Statistical-chain-based dynamic polymer theory

Experimental and simulation results

Figure S1 to S31

References

Other Supplementary Materials for this manuscript include the following:

Video S1

1. Synthesis:

Synthesis of crosslinker 1,4-phenylenebis(methylene) bis(cyclopent-3-ene-1-carboxylate)

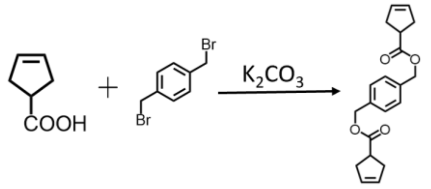

The crosslinker was synthesized following the procedure in literature.[1] K_2CO_3 (4.93 g, 35.7 mmol, 5.0 molar equivalents) was mixed with acetone (30 mL), followed by adding cyclopent-3-enecarboxylic acid (2 g, 17.8 mmol, 2.5 molar equivalents). Then α,α′-dibromo-*p*-xylene (1.88 g, 7.13 mmol, 1.0 molar equivalents) was added to the mixture and reacted at 60 °C for 12 hours. The reaction mixture was then filtered, and the filtrate was concentrated. 20 mL of ethyl acetate was then added to the concentrated residue and washed with brine, followed by drying with anhydrous sodium sulfate and then concentrated. The concentrate was then purified by flash chromatography with ethyl acetate and hexane (5:100) to give the crosslinker as a colorless oil (1.74 g, 75%). ^{1}H-NMR ($CDCl_3$, 400 MHz) δ 7.35 (s, 4 H), 5.67 (s, 4 H), 5.13 (s, 4 H), 3.17 (m, 2 H), 2.68-2.66 (m, 8 H).

Synthesis of catalyst cis-Ru-1

The synthesis of cis-Ru-1 was carried out following the procedure reported in the literature.[2] Grubbs G3 (0.2 g, synthesized following literature[3]) was dissolved in 10 mL of anhydrous dichloromethane (DCM) under an argon atmosphere. To this solution, tribenzyl phosphite (0.194 g, 0.55 mmol) ligand was added, resulting in an immediate color change from green to dark brown. The reaction mixture was stirred at room temperature for 2 hours. Subsequently, the solvent was removed under reduced pressure, and the residue was redissolved in a minimal amount of anhydrous DCM. An excess of pentane was added and the mixture was cooled to -20 °C for 2 hours to induce precipitation. The resulting solid was collected, redissolved in a minimal amount of toluene, and pentane was added dropwise until the solution turned cloudy. This mixture was stored at -20 °C for a few days to allow crystallization. The cis-Ru-1 was obtained as purple crystals (20 mg, 10% yield). The structure was confirmed by ^{1}H-NMR spectroscopy, which matched the previously reported data,[2] as shown in **Figure S30**.

2. Statistical-chain-based dynamic polymer theory

The theory is adapted from this work.[4] We summarize its fundamental aspects in a dimensionless form here.

The dynamic “living” polymer system consists of three components: the polymer network, imbibed monomers, crosslinkers and catalysts. The polymer network is characterized by three key variables: chain density C, segment density C_n, and chain distribution tensor $\boldsymbol{\gamma}$. The reference concentrations of the monomers and crosslinkers are denoted as C_m and C_{cr}, respectively. Time is nondimensionalized using the forward reaction rate $k_p C_{ra}$, where k_p is the forward reaction rate

constant and C_{ra} is the catalyst concentration. Length is normalized by the characteristic size of the gel.

Four reactions are considered in the "living" polymer system: chain insertion, new chain formation, chain extraction, and chain exchange. The rates of these reactions are denoted as Π_{in}, Π_{new}, Π_{ex}, and $\Pi_{exchange}$, respectively. Their dimensionless reaction rates are given by:

$$\Xi_{in} = (1 - r_{new})C_m/J \tag{1}$$

$$\Xi_{new} = r_{new}C_m/J \tag{2}$$

$$\Xi_{ex} = k_{ex}C_n/J \tag{3}$$

$$\Xi_{exchange} = k_d/J \tag{4}$$

Compared to our previous work[4], only Equation (3) differs, where C_n replaces C. The proportion of monomers that form new chains is calculated by $r_{new} = C_{cr}/(\alpha C + C_{cr})$, where α is a phenomenological parameter controlling the balance between chain insertion and new chain formation. Here, k_{ex} and k_d are the dimensionless reaction constants for chain extraction and chain exchange, respectively, representing the ratio of their reaction rates to the forward reaction rate. In experiments, the reaction rate constant can be complex due to the presence of an induction time, and the changing conditions of the surrounding solutions, which could lead to inhomogeneous reaction rate throughout the sample. For simplicity, we assume a homogeneous effective reaction rate constant with specific values fitted for each experiment. As a result, the reaction rate only vary with catalyst and monomer concentrations. Here, $J = \det \mathbf{F}$ is the swelling ratio of the dynamic "living" polymer relative to its dry state, where $\mathbf{F}$ is the deformation gradient.

Thus, the evolution equations governing the network variables are

$$\frac{\mathrm{d}C}{\mathrm{d}t} = r_{new}\frac{f}{2}\frac{C_{cr}}{J} \tag{5}$$

$$\frac{\mathrm{d}C_n}{\mathrm{d}t} = \frac{C_m - k_{ex}C_n}{J} \tag{6}$$

$$\frac{\mathrm{d}\boldsymbol{\gamma}}{\mathrm{d}t} = -\frac{2}{3}\frac{(1 - r_{new})C_m - k_{ex}C_n}{C_n J}\boldsymbol{\gamma} + \left(r_{new}\frac{f}{2}\frac{C_{cr}}{CJ} + \frac{k_d}{J}\right)(\mathbf{I} - \boldsymbol{\gamma}) + \mathbf{L}\cdot\boldsymbol{\gamma} + \boldsymbol{\gamma}\cdot\mathbf{L}^T \tag{7}$$

where $\mathbf{L} = \dot{\mathbf{F}}\mathbf{F}^{-1}$ is the velocity gradient, f is the coordination number of the polymer network, and $\mathbf{I}$ is the second order identity tensor.

The governing equations of the dynamic "living" polymer include the force balance, and mass concervation:

$$\mathrm{Div}\mathbf{P}^T = 0 \tag{8}$$

$$\frac{\mathrm{d}C_m}{\mathrm{d}t} + \mathrm{Div}\mathbf{J}_m = \frac{k_{ex}C_n - C_m}{J} \tag{9}$$

$$\frac{\mathrm{d}C_{cr}}{\mathrm{d}t} + \mathrm{Div}\mathbf{J}_{cr} = -r_{new}\frac{C_{cr}}{J} \tag{10}$$

where $\mathbf{P}$ is the first Piola-Kirchhoff stress, neglecting body force, and $\mathbf{J}_m$ and $\mathbf{J}_{cr}$ are the monomer flux and crosslinker flux, respectively.

The stress-strain relations is given as

$$\mathbf{P} = C(\boldsymbol{\gamma} - \mathbf{I})\mathbf{F}^{-\mathrm{T}} - \Pi J \mathbf{F}^{-\mathrm{T}} \tag{11}$$

where Π is the osmotic pressure, which enforces the incompressibility constraint $J = C_m + C_n$, meaning the volume change of the polymer is due to the change of the monomer and crosslinker contents.

The fluxes of the monomers and crosslinkers adopt Fick's law:

$$\mathbf{J}_m = -D_m C_m \mathbf{F}^{-1} \cdot \mathbf{F}^{-\mathrm{T}} \cdot \frac{\partial \mu_m}{\partial \mathbf{X}} \tag{12}$$

$$\mathbf{J}_{cr} = -D_m C_m \mathbf{F}^{-1} \cdot \mathbf{F}^{-\mathrm{T}} \cdot \frac{\partial \mu_{cr}}{\partial \mathbf{X}} \tag{13}$$

where μ_m and μ_{cr} are chemical potentials of monomers and crosslinkers, respectively, and are given by

$$\mu_m = \ln \frac{C_m}{J} + \frac{C_n}{J} + \frac{\chi C_n^2}{J^2} - \frac{C_{cr}}{J} + \Pi \tag{14}$$

$$\mu_{cr} = \ln \frac{C_{cr}}{J} \tag{15}$$

Simulation of the growth behaviors under different reaction mechanisms (Figure 1a)

To generate the growth curves in Figure 1a, different parameters are applied. The pure swelling curve assumes that all reaction rates are zero. For the new chain formation, $k_p C_{ra}$ becomes nonzero, but α is set to zero to ensure that only new chain formation occurs. To add the chain exchange, k_d is set to nonzero. To incorporate chain insertion, α is made nonzero so that both new chain formation and chain insertion are present. All of these curves require monomer diffusion from the outside, so μ_m is set to 0. For degrowth, no monomers are present outside the gel. μ_m is set to -2 to reflect this condition.

Simulation of the pure swelling behavior of the "living" polymer without catalyst

For pure swelling of the thin film, the 3D geometry is reconstructed based on experimental condition. The initial swelling ratio of the polymer is 1.42. To simulate immersion in a monomer solvent, the chemical potential of the monomer μ_m at the boundary is set to zero. All reaction rates are set to zero, ensuring that swelling is driven solely by monomer diffusion. By fitting the swelling ratio of the thin film as a function of time, we obtain a monomer diffusivity of $D_m = 5.0 \times 10^{-9}$ m^2/s for the thin film. Similarly, for the cubic gel, simulation yields $D_m = 4.0 \times 10^{-9}$m^2/s. The slight difference in diffusivity may result from sample-to-sample variations.

Simulation of homogeneous growth of thin films (Figure 2b(ii))

The initial conditions are the same as in the pure swelling case. At $t = 0$, μ_m is set to zero, and the concentration of the crosslinker is set to $C_{cr}/J = 0.02$ based on the experimental recipe. The overall mass and dry mass of the film as functions of time are used to fit parameters $k_p C_{ra}$, k_d and

α. The resulting values are $k_p C_{ra} = 1.20 \times 10^{-5}\mathrm{s}^{-1}$, $k_d = 4.30$ and $\alpha = 1.0$. As shown in Figure 2b(ii), the simulation results on both dry mass and wet mass of the "living" polymer during the growth process agree well with the experiment results.

Simulation of inhomogeneous growth of the cubic samples (Figure 2c(ii))

The growth simulation of the cubic polymer is divided into two states. In the first growth stage, the sample grows under the same conditions as the thin films. It evolves for 3 hours under coupled diffusion and reaction. In the second drying stage, all reactions are halted, μ_m is set to -2 to simulate the drying process, and C_{cr} is set to zero at the boundary. The simulation continues until the overall monomer concentration inside the "living" polymer C_m falls below 0.001, indicating complete drying. To expedite the simulation, a 2D axisymmetric model is used to represent a cylindrical sample with a height and diameter matching the cube's edge length. The mass changes in the cylindrical and cubic models are found to be similar, validating this simplification. The values of k_d and α are taken from the thin film simulations, with the only fitting parameter being $k_p C_{ra} = 2.79 \times 10^{-5}\mathrm{s}^{-1}$. As shown in Figure 2c(ii), the simulation results on both dry mass and wet mass of the cubic "living" polymers during the growth process agree well with the experiment results.

Simulation of degrowth of the cubic samples (Figure 2d (ii))

In the degrowth simulation, the polymer starts with an initial swelling ratio of $J = 1.42$ and contains no free crosslinkers. The μ_m is set as -2, and C_{cr} is kept at zero at the boundary. During degrowth, the polymer remains close to the dry state, causing the monomer diffusivity to be significantly lower than in the swollen state. Additionally, the concentration of the catalyst C_{ra} gradually decreases over time, as no new catalyst is supplied from the external environment. To model this, we assume an exponential decay, $C_{ra} = C_{ra0}e^{-kt}$, where k is the decay factor and C_{ra0} is the initial catalyst concentration. By fitting the simulation to experimental results, the following parameters are obtained: $D_m = 3.2 \times 10^{-11}$ m^2/s, $k_p C_{ra0} = 2.09 \times 10^{-4}\mathrm{s}^{-1}$ and $k = 3.2 \times 10^{-5}\mathrm{s}^{-1}$.

Simulation of light-induced surface microstructure growth

The swollen polymer is modeled as a cylinder with a radius and thickness of 3 mm. The material parameters used are the same as those in the simulation of the growth of the dynamic "living" polymer. The top middle part of the sample is activated with reaction while other parts do not have reactions. The activated region is set based on the experiment. Based on experimental observations, the light penetration depth is estimated to be around 100 μm, and the radius of the reaction layer corresponds to the light beam size.

3. Experimental and simulation results

“Seed” to “Tree” growth experiment. A mold with a tree-shaped groove was fabricated via 3D printing using an ABS-like resin. A narrow hole was drilled into the bottom side wall to allow needle access. The “seed” polymer was synthesized by polymerizing a 100 μL solution containing 99.5 vol.% CP-ester, 0.5 vol.% crosslinker, and 15 mg/mL G2. The resulting seed was then placed at the bottom of the mold. A glass sheet was positioned over the mold and clamped tightly. To support polymer growth, monomer solution of the same composition was injected into the seed polymer through the bottom side channel using a syringe needle, with 50 μL added every 1.5 hours. Approximately 50 such injections were required to fully form the tree-like structure. Notably, the monomer solution was injected directly into the seed polymer, not to fill the entire mold chamber.

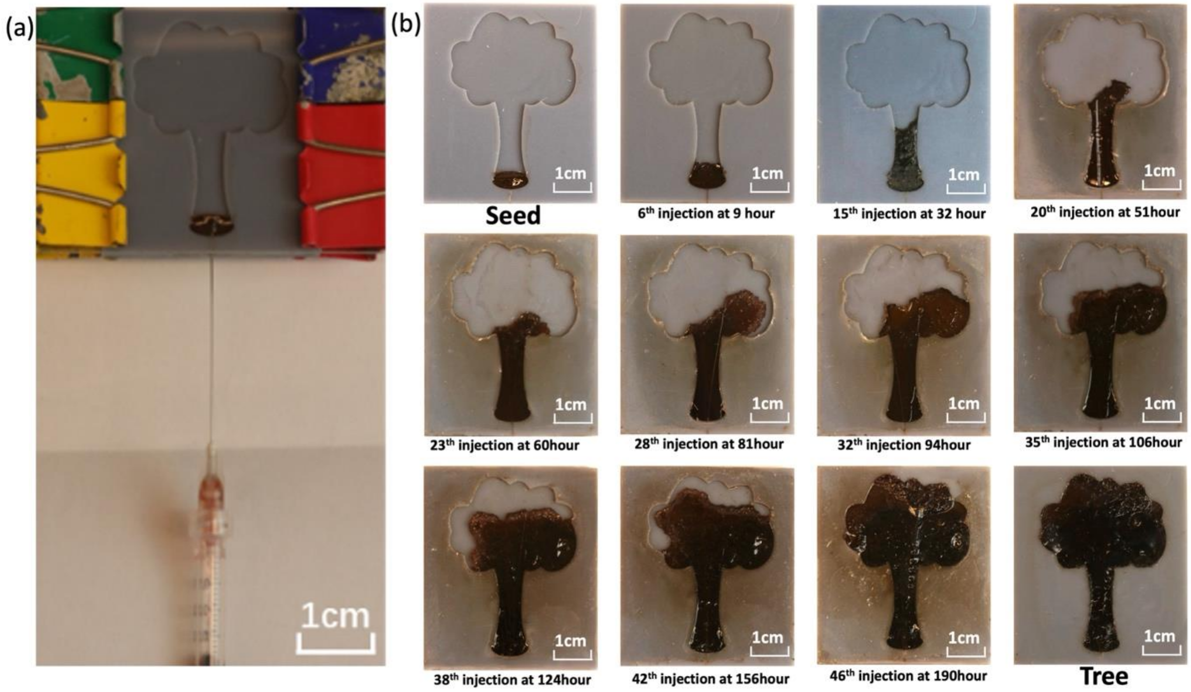

Figure S1. **a** Experimental setup for the “seed” to “tree” experiment. **b** The growth process of the seed in the mold with continuous supply of nutrient solution to the growing polymer.

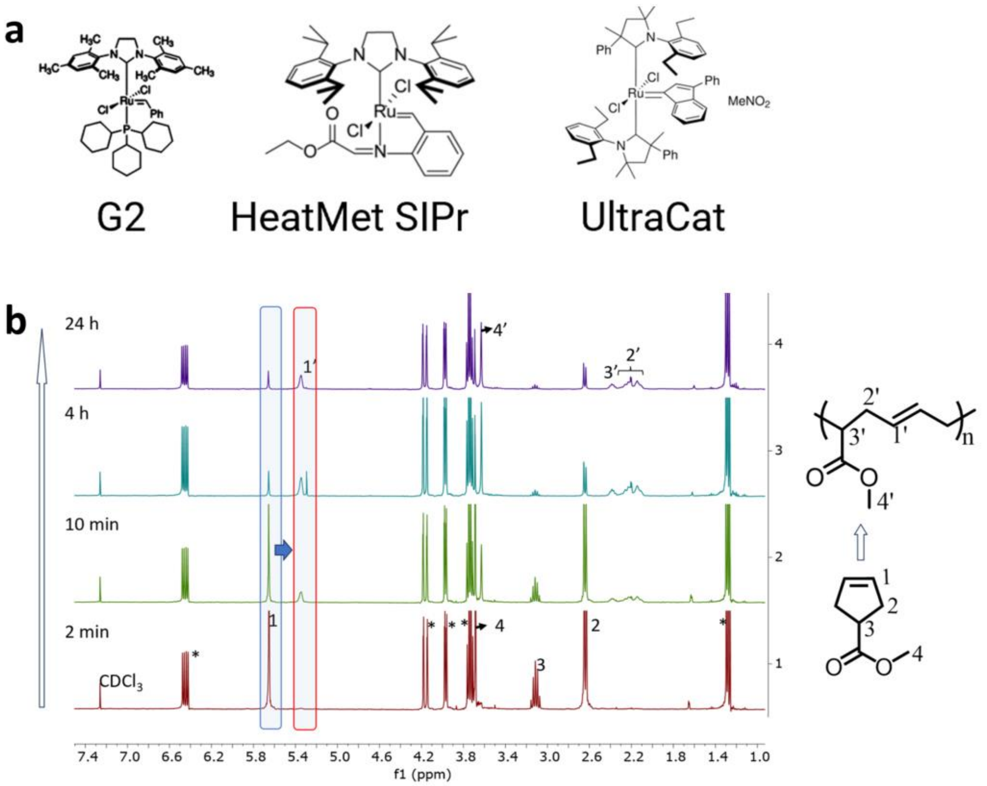

Figure S2. **a** Chemical structures of thermal catalysts explored for controlling polymerization kinetics. **b** ^{1}H-NMR spectra of G2 (20 mg/mL) catalyzed polymerization of CP-ester for different time, with * highlighting the peaks from ethyl vinyl ether (EVE).

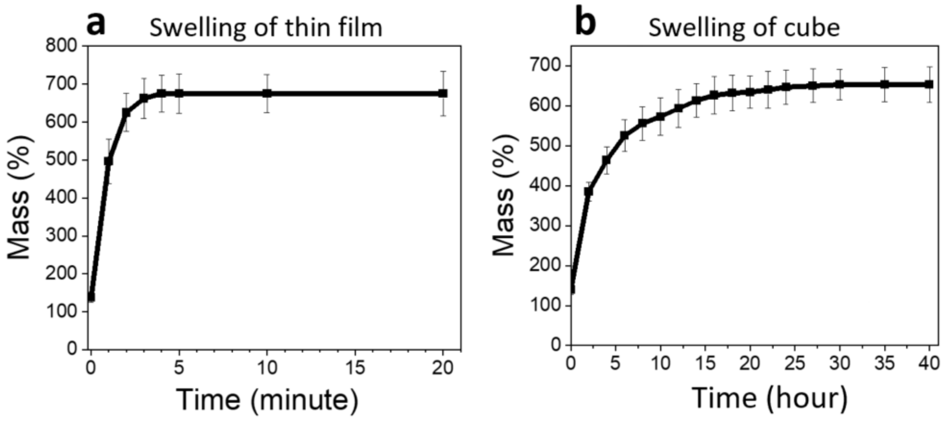

Figure S3. Pure swelling behavior: **a** Pure swelling of a deactivated thin film (8 × 8 × 0.12 mm³; polymerized from 98 vol.% CP-ester, 2 vol.% crosslinker, 2.5 mg/mL G2, TCHP:G2 = 31:1) in a monomer solution composed of 95 vol.% (98% CP-ester, 2% crosslinker, 2.5 mg/mL G2, TCHP:G2 = 31:1) and 5 vol.% EVE. **b** Pure swelling of a polymer cube (5.3 × 5.3 × 5.3 mm³; polymerized from 98 vol.% CP-ester, 2 vol.% crosslinker, 7 mg/mL G2 for 3 hours) in a similar monomer solution composed of 95 vol.% (98% CP-ester, 2% crosslinker, 7 mg/mL G2) and 5 vol.% EVE.

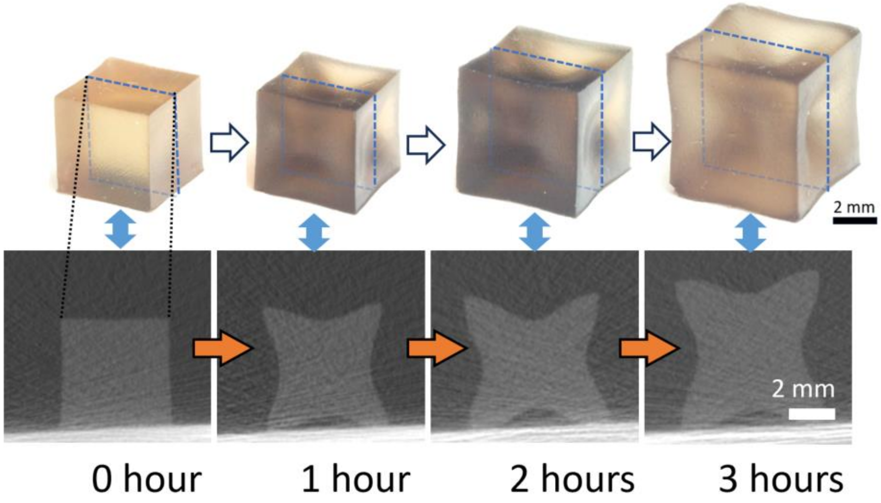

Figure S4. Cross-section images of the grown polymer cubes (wash, dried) after growing in the monomer solution (98% CP-ester, 2% crosslinker, 7 mg/mL G2) for varying time, obtained via micro-computed tomography.

Micro-computed tomography (micro-CT) scan. Surface profiling of the polymer cubes was conducted using a Scanco μCT50 imaging system (SCANCO Medical, Brüttisellen, Switzerland). Samples were placed in a cylindrical holder (34 mm diameter) and fixed on the carousel sample stage. Scanning was performed using an X-ray energy of 45 kV and a current of 133 μA, with a 0.1 mm aluminum filter. The integration time was set to 300 milliseconds, and the voxel size was 48.6 μm. Reconstructed three-dimensional images were generated using the DICOM viewer software provided by the manufacturer.

Growth performance under different reaction kinetics and crosslinker density: In **Figure S5**, all samples for growth start from the same size ($0.12 \times 2.2 \times 2.2$ mm^3), and the monomer solutions used for growth of the thin films are identical to the ones used to mold the starting thin films, with more details in experimental section. **Figure S5a** shows the influence of reaction kinetics on growth. The different reaction kinetics was controlled by tuning the molar ratio of TCHP : G2 while keeping the same G2 concentration of 2.5 mg/mL, with the corresponding polymerization kinetics shown in **Figure 2a**. **Figure S5a(iii)** shows that faster reaction kinetics (lower TCHP: G2 ratio) resulted in a larger mass increase after the same time, which makes sense as more monomers are polymerized and integrated into the original network after the same time. Also, all these samples show uniform growth, because these thin films have timescale of swelling smaller than reaction timescale, the same reason as discussed in the main text. Additionally, as shown in **Figure S5b**, higher crosslinker density in the film and the monomer solution induced smaller growth. This may be because that higher crosslinker density leads to a polymer network with smaller diffusivity, lower swell ratio and higher rigidity that makes the network less efficient in chain exchange to incorporate the swollen monomer solution into the network.

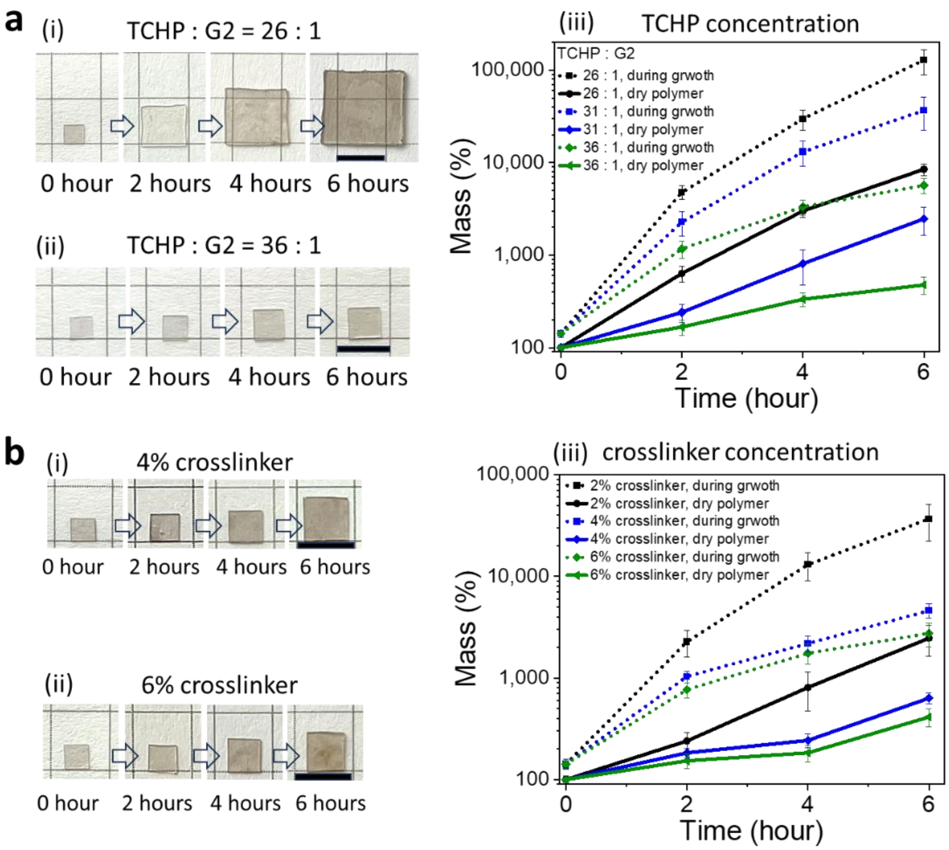

Figure S5. Growth behavior under varying reaction kinetics and crosslinker concentrations. a Influence of reaction kinetics on growth: (i) Images of four poly(CP-ester) thin films (2 vol.% crosslinker) after growth in monomer solution (98 vol.% CP-ester, 2 vol.% crosslinker, 2.5 mg/mL G2, TCHP:G2 = 26:1), followed by G2 deactivation, washing, and drying. (ii) Same as (i), but with a TCHP:G2 molar ratio of 36:1. (iii) Mass changes of the films over time during growth for samples with varying TCHP:G2 ratios. For the ones labeled "during growth", mass was measured without deactivating G2 or performing washing and drying steps. For the ones labeled "dry polymer", mass was measured after deactivating G2, washing, and drying. **b** Influence of crosslinker concentration on growth: (i) Images of four poly(CP-ester) thin films with 4 vol.% crosslinker after growth in monomer solution (96 vol.% CP-ester, 4 vol.% crosslinker, 2.5 mg/mL G2, TCHP:G2 = 31:1), followed by deactivation, washing, and drying. (ii) Images of four poly(CP-ester) thin films with 6 vol.% crosslinker under similar conditions (94 vol.% CP-ester, 6 vol.% crosslinker). (iii) Mass changes of the films during growth for samples with varying crosslinker concentrations. The labeling of "during growth" and "dry polymer" has the same meaning as **FigureS5a(iii)**. Scale bars are all 5 mm.

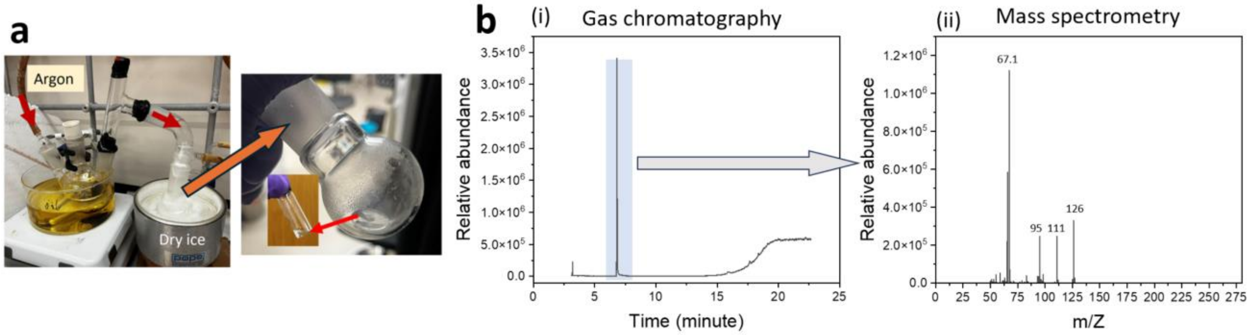

Figure S6. Recycling the poly(CP-ester). a Picture of the setup used to heat poly(CP-ester) (96 vol.% CP-ester, 4 vol.% crosslinker, 10 mg/mL G2) to 35 °C, initiating degradation and enabling the collection and recycling of evaporated components. **b** The (i) gas chromatography and (ii) mass spectrometry of the recycled liquid from the setup.

Degrowth performance under varying reaction kinetics and crosslinker densities: Degrowth experiments were carried out by placing freshly prepared thin films (~1 mm × 5 mm × 5 mm)—immediately after polymerizing CP-ester with the crosslinker for 5 hours—into a container under an argon flow (~36 L/hour, **Figure S7a**). This flow removes the released monomer, thereby driving concurrent depolymerization, monomer diffusion and evaporation, and chain exchange, all contributing to the degrowth process. **Figure S7b** illustrates the shape change and mass loss of the films during degrowth. Most samples exhibited uniform degrowth, with the exception of the sample containing 2% crosslinker. In this case, degrowth-induced depolymerization and chain exchange significantly weakened the network, resulting in insufficient crosslinking. Consequently, the film sagged under gravity and displayed degradation-like behavior after 5 days. As shown in **Figure S7c**, increasing the crosslinker density led to slower mass loss and a higher remaining mass ratio. This is attributed to the fact that crosslinkers do not evaporate like monomers under the experimental conditions. As demonstrated in **Figure S6**, only CP-ester monomer is recycled even at elevated temperature of 35 °C. The CP-ester monomer used to synthesize the polymer was recovered at 53.78% ± 6.85%, though the recovery could theoretically approach ~90%, as suggested by the mass change in Figure 2d(ii), if all recycled chemicals were successfully collected. The lower experimental recovery mainly results from limitations in the current setup: a portion of the evaporated chemicals is carried out of the collection bottle by the Ar flow (~5 mL/min). Although the Ar flow is necessary to transport the evaporated species into the cooled collection bottle, it continues to vent afterward, causing some material loss. A higher recovery efficiency could be achieved by modifying the apparatus to include a longer cooling pathway before venting the Ar–monomer stream and by using a lower condensation temperature. The present setup was originally designed to analyze the composition of recycling products rather than to quantify recycling efficiency. Nonetheless, this measurement confirms that only CP-ester is released from the system and that the recorded mass accurately represents the true amount of monomer recovered. Additionally, the reduced mass loss rate with higher crosslinker content is likely due to the formation of a denser and less flexible polymer network, which restricts monomer diffusivity and catalyst mobility, thereby slowing the reaction kinetics. **Figure S7d** shows that increasing the G2 catalyst concentration, which accelerates reaction kinetics, resulted in a higher rate of mass loss—particularly within the first 24 hours. After this period, the mass loss rate and the remaining mass plateaued, likely due to the limited amount of residual poly(CP-ester) available for further depolymerization.

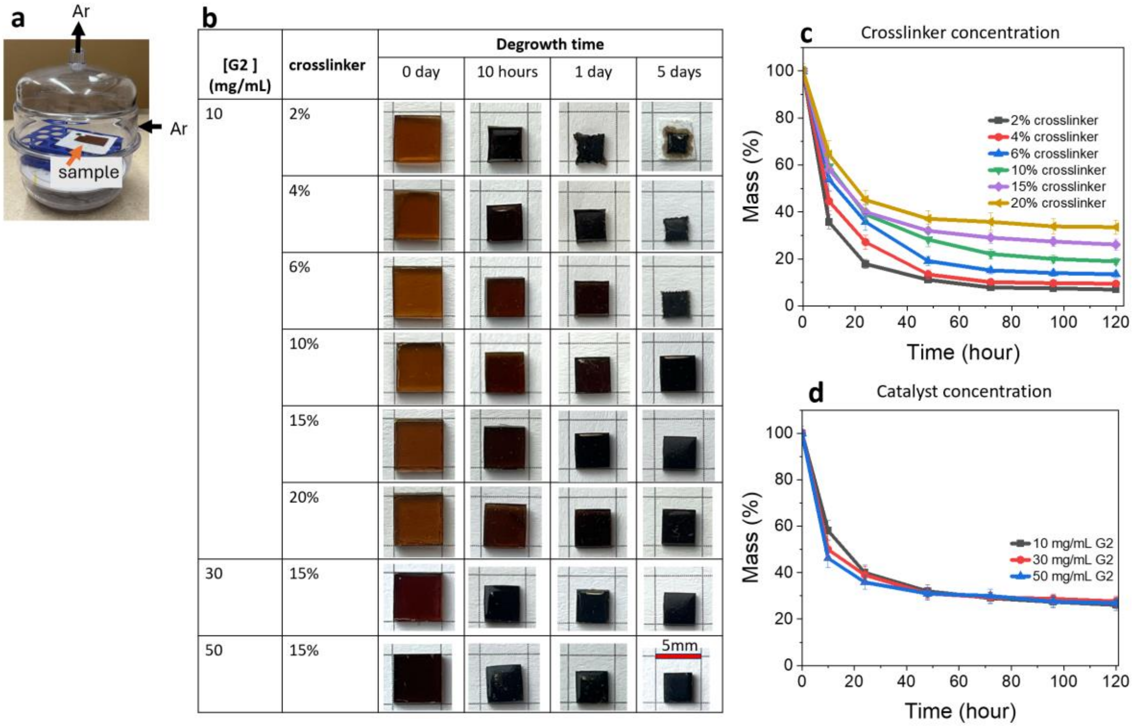

Figure S7. Degrowth/degradation behaviors. **a** The experimental setup for ambient-temperature degrowth of 1 mm-thick poly(CP-ester) films at 22 °C, using an argon (Ar) flow (~36 L/hour) to carry away the extracted monomers. **b** Images documenting the degrowth process of thin films with an initial size of approximately 1 mm × 5 mm × 5 mm. **c** Mass change of thin films over time during degrowth, for samples with varying crosslinker concentrations (all containing 10 mg/mL G2). Mass was measured without deactivating G2 or performing washing and drying steps. **d** Mass change of thin films during degrowth for samples with varying catalyst (G2) concentrations, all with a fixed crosslinker content of 15 vol.%. Mass was measured without deactivating G2 or performing washing and drying steps.

Photocatalyst screening: To identify an effective photocatalyst for precise control of localized growth and degradation as discussed in the main text, we screened the catalysts shown in **Figure S8a**. The selection of Benzil and ITX as photosensitizers and EDAB as a co-initiator was inspired by the work of photo-initiated ROMP in literature.[5] An optimal photocatalyst should have a few key properties: (1) exhibit minimal reactivity with the CP-ester monomer in the dark, (2) be efficiently activated by 405 nm light, and (3) enable rapid and effective initiation of CP-ester polymeriztion once activated. To assess dark reactivity, mixtures of CP-ester and each catalyst were stored in the dark, and gel time was measured by periodically inverting the vial. Among the tested catalysts, HeatMet showed undesirable performance, showing CP-ester gelation within a few hours (**Figure 8b**), while the others demonstrated significantly longer gel times, indicating greater stability in the absence of light. Next, we quantified the efficiency of photo-initiated polymerization under 405 nm irradiation. Mixtures of CP-ester and catalyst were irradiated for a certain duration, and monomer conversion was subsequently measured via ^{1}H-NMR (**Figure S8c**). After testing various catalyst systems (**Figure S8c(i–iii)**), cis-Ru-1 emerged as the most effective catalyst for CP-ester polymerization (**Figure S8c(iii)**). To assess catalyst stability post-irradiation, we tested whether cis-Ru-1 remained active after light exposure (**Figure S8c(iv)**). Remarkably, just 5 minutes of 405 nm light exposure was sufficient to activate cis-Ru-1, which then continued to drive polymerization even in the absence of light. The final monomer conversion reached ~72%, comparable to that induced by G2, highlighting the robustness and efficiency of cis-Ru-1. Additionally, cis-Ru-1 exhibited minimal reactivity with CP-ester in the dark even after 70 hours

(**Figure S8c(v)**). Taken together, these results identify **cis-Ru-1** as the optimal photocatalyst: it is stable in the dark, rapidly and efficiently activated by 405 nm light, and remains active post-irradiation to drive polymerization to equilibrium.

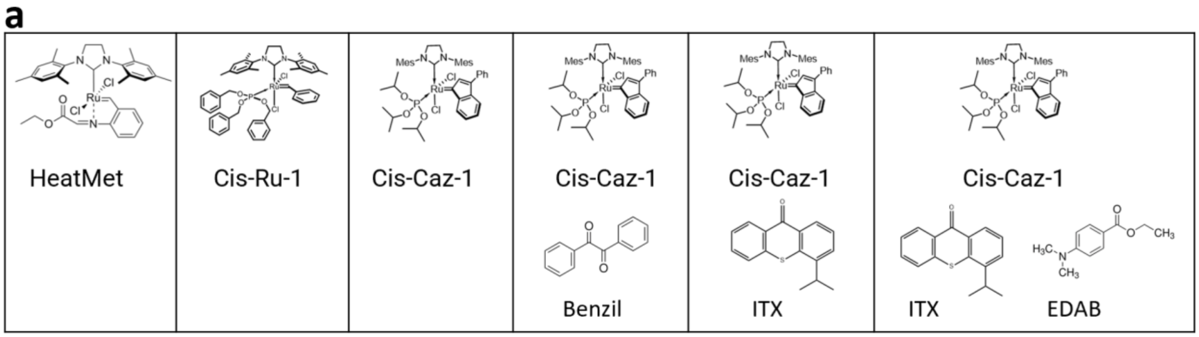

b

Catalyst	Gel time in dark
HeatMet	~4 h
Cis-Caz-1	No gel after 7 days
Cis-Caz-1 + Benzil	No gel after 7 days
Cis-Caz-1 + ITX	No gel after 7 days
Cis-Caz-1 + Benzil+ EDAB	No gel after 7 days
Cis-Caz-1 + ITX + EDAB	No gel after 7 days
Cis-Ru-1	No gel after 2 days

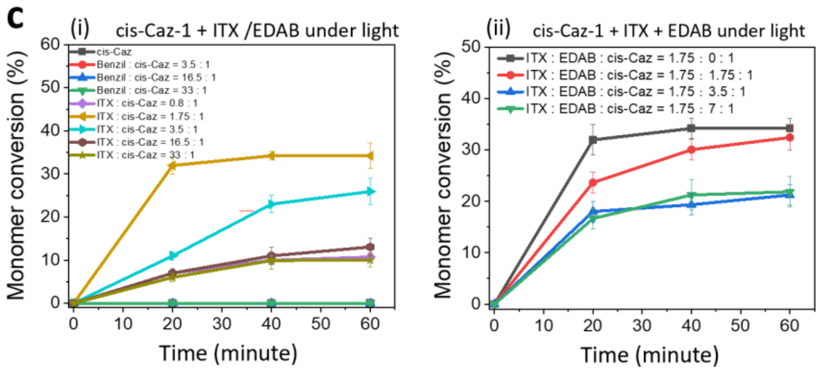

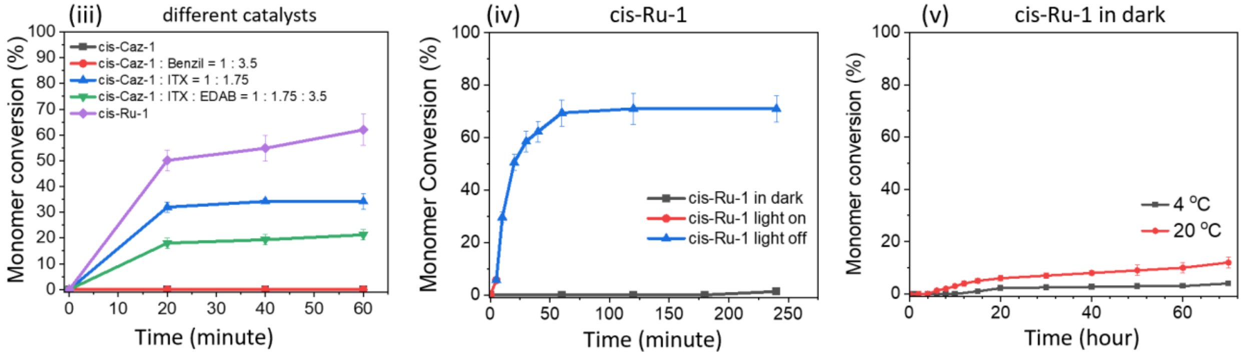

Figure S8. Photocatalyst screening. **a** A set of catalysts screened for 405 nm light (200 mW/cm²)-initiated polymerization of CP-ester. **b** Gel time of a mixture containing catalyst (10 mg/mL), 98 vol.% CP-ester, and 2 vol.% crosslinker, measured in the dark at room temperature (~22 °C) as the time required for the mixture to stop flowing when the vial is inverted. **c** The CP-ester monomer conversion over time of light irradiation measured by ^{1}H-NMR, (i) Polymerization catalyzed by cis-Caz-1 (10 mg/mL) with photosensitizers Benzil or ITX. (ii) Polymerization catalyzed by cis-Caz-1 (10 mg/mL) in combination with ITX and the co-initiator EDAB. (iii) Summary of maximum monomer conversion for different catalyst systems: cis-Caz-1 alone, cis-Caz-1 with Benzil, cis-Caz-1 with ITX, cis-Caz-1 with ITX and EDAB, and cis-Ru-1 (all at 10 mg/mL). (iv) CP-ester monomer conversion catalyzed by cis-Ru-1 (10 mg/mL) under three conditions: in the dark, under 5 minutes of 405 nm light exposure, and in the dark after the 5-minute light activation. (v) CP-ester monomer conversion catalyzed by cis-Ru-1 (10 mg/mL) in the dark at two temperatures: 4 °C and 22 °C.

Light-induced local growth

Micro-pillar width change during growth under varying catalyst concentrations and light intensities: The concentration of the cis-Ru-1 catalyst in the monomer solution and the applied light intensity both affect the effective concentration of photo-activated catalyst available to initiate polymerization. As shown in **Figure S9a**, the width of the micro-pillars increases slightly over time and can slightly exceed the light irradiation diameter of 500 µm. This is likely due to the sustained activity of the light-activated catalyst, which continues to polymerize monomer that diffuses into the irradiated region—even slightly beyond the nominal light exposure area. However, the overall pillar width remains close to the irradiation diameter. Higher catalyst concentrations result in marginally wider pillars, likely due to increased polymerization efficiency. Additionally, increasing light intensity leads to more significant increases in pillar width, with a notable difference observed between 200 mW/cm² and 1600 mW/cm² (**Figure S9b**). This is attributed to the higher concentration of activated catalyst generated at elevated light intensities.

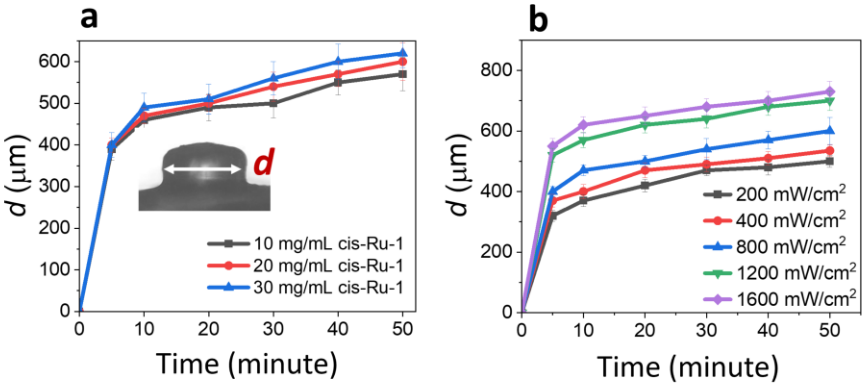

Figure S9. Micro-pillar width evolution during growth under varying catalyst concentrations and light intensities. a Half-height width (*d*) of grown micro-pillars as a function of irradiation time for samples composed of 97 vol.% CP-ester and 3 vol.% crosslinker, swollen with monomer solutions containing varying concentrations of *cis*-Ru-1 (98 vol.% CP-ester, 2 vol.% crosslinker). Irradiation was performed with 405 nm light (500 µm diameter, 800 mW/cm²). **b** Half-height width (*d*) of grown micro-pillars as a function of irradiation time for samples (97 vol.% CP-ester, 3 vol.% crosslinker) swollen with a monomer solution containing 98 vol.% CP-ester, 2 vol.% crosslinker, and 20 mg/mL *cis*-Ru-1, under varying light intensities. All experiments used a light spot diameter of 500 µm.

Influence of photo-thermal effect on growth: When the light intensity is further increased to 3200 mW/cm^2, the pillars become shorter (**Figure S10a**). The cross-section views of the pillars (**Figure S10b**) show a smaller height in the center than the height near the edges, which can be attributed to the photo-thermal effect that induced more monomer evaporation in the center than the edges, as the edges have lower temperature through heat conduction to the surroundings. The temperature under light irradiation is then quantified with a thermal camera (**Figure S10c**), proving that the center of light irradiation has a higher temperature than the edges. Also, the temperature at the center of light irradiation increases with increasing light intensity (**Figure S10c(ii)**). The temperature at the center of light irradiation also slightly increased with increasing catalyst concentration, as catalyst's light absorption is the main reason of such photo-thermal effect (**Figure S5d**).

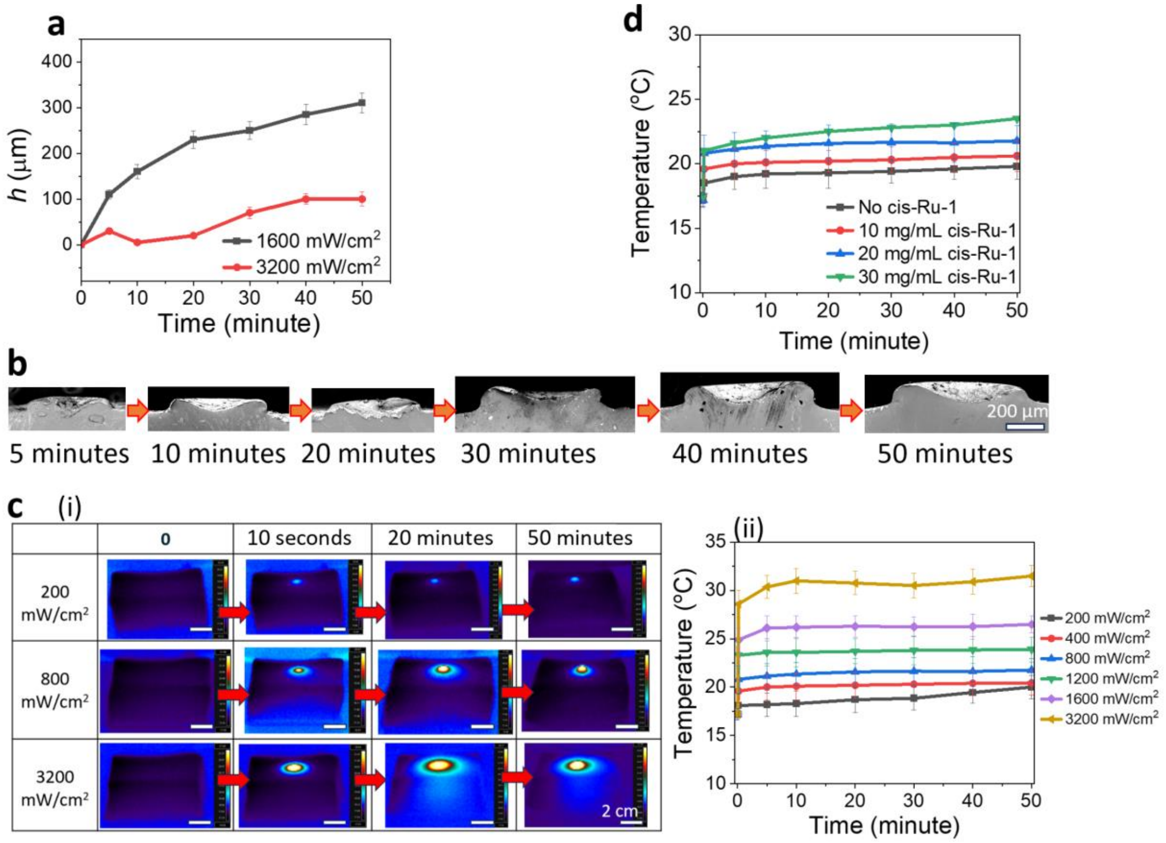

Figure S10. Photothermal effects during micro-pillar growth. a Central height (h) of grown micro-pillars as a function of irradiation time under different light intensities. **b** SEM cross-sectional images of micro-pillars grown for various durations under 405 nm light at 3200 mW/cm², showing morphological evolution. **c** (i) Thermal camera images showing temperature distribution over time on samples irradiated with 405 nm light (500 µm diameter) at different intensities. The temperature scale ranges from low (dark colors) to high (bright colors). (ii) Temperature evolution at the center of the irradiated region over time, under 405 nm light (500 µm diameter) at varying intensities. **d** Temperature change at the center of the irradiated area (405 nm, 500 µm diameter, 800 mW/cm²) over time for poly(CP-ester) samples swollen with monomer solutions containing different concentrations of cis-Ru-1.

Influence of crosslinker density on light-induced local growth: Higher crosslinker density in the sample and the monomer solution leads to a smaller height after the same time of growth (**Figure S11a**). This can be attributed to the more rigid network when highly crosslinked, which could decrease the reaction rate and diffusivity of monomer solution. Also, the polymer's swell ratio is smaller with higher crosslinker density (**Figure S11c**), which reduces the amount of available monomer solution for diffusion and growth. The pillar width d is only slightly smaller for (**Figure S11b**) for a sample of higher crosslinker density as the width is mainly dominated by light dot size.

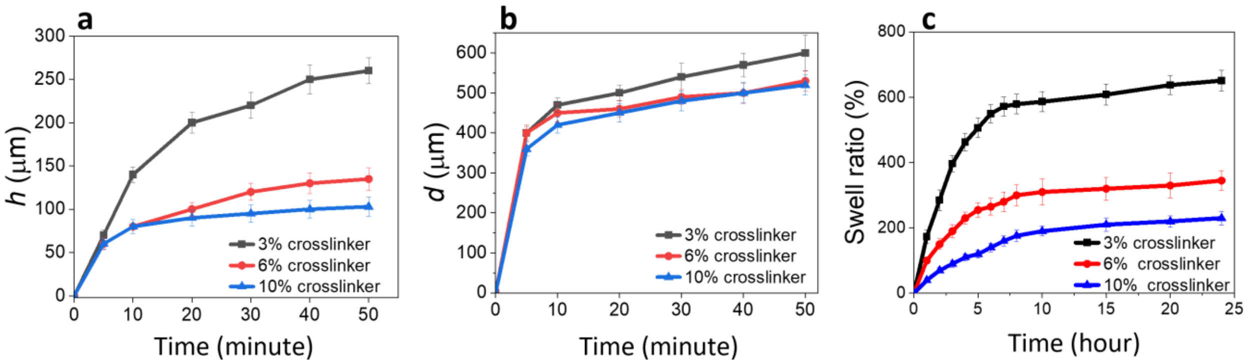

Fig S11. Light-induced local growth under different crosslinker densities: The height (**a**) and width (**b**) of grown micro-pillars as a function of irradiation time (light: 405 nm, 500 μm diameter, 800 mW/cm^2) for samples and monomer solution with different concentrations of crosslinker, all with 20 mg/mL cis-Ru-1. Note the polymer samples and the monomer solution have the same crosslinker density. **c** The swelling ratio (defined as $(m_t\text{-}m_0)/m_0$) of samples of varying crosslinker density after immersing in a monomer solution (with the same concentrations of crosslinker as that used in making the elastomer, all with 20 mg/mL cis-Ru-1) at 4 °C. The m_t is the mass at the time of measurement and m_0 is the original mass of the film before swelling.

Influence of light irradiation area on growth: As shown in **Figure S12**, reducing the light beam diameter led to decreased pillar height and narrower width. Pillars grown under 500 μm and 1000 μm light spot sizes exhibited comparable heights, both significantly greater than those formed under a 50 μm beam. This discrepancy may be attributed to mechanical constraints imposed by the surrounding polymer network, where no chain exchange occurs. Smaller light-irradiated areas experience greater restriction forces per unit area from the adjacent unreacted matrix, limiting vertical growth. Across all beam sizes, the resulting pillar width closely matched the diameter of the corresponding light spot.

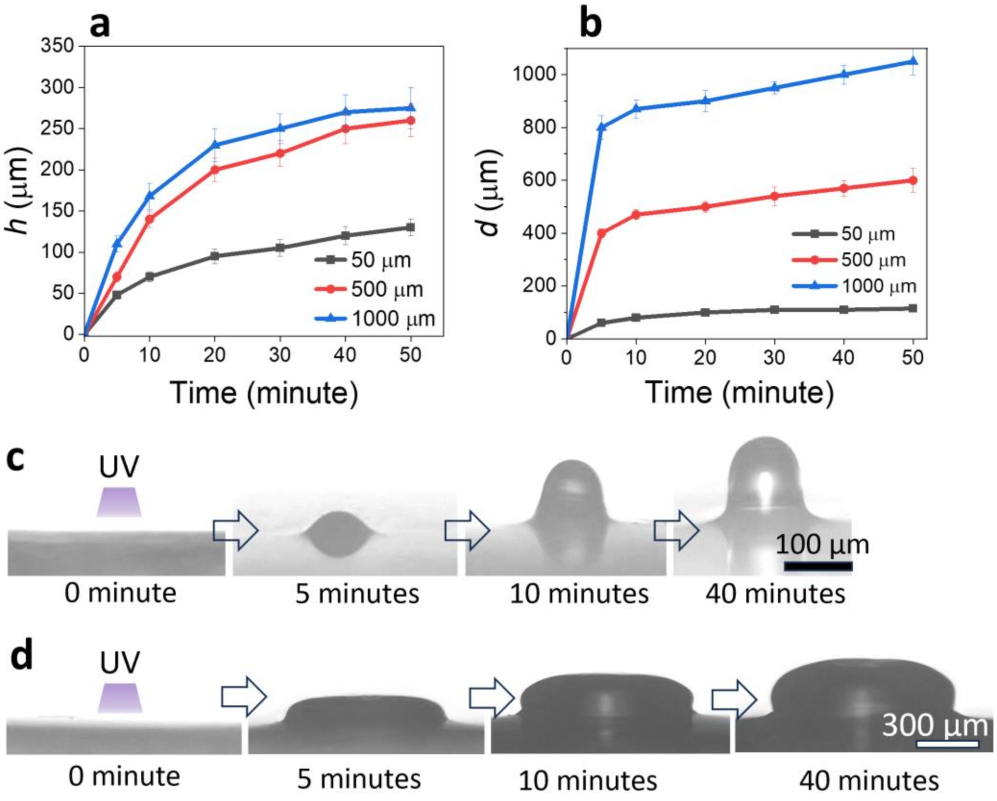

Fig S12. Growth under varying light irradiation size: The height (**a**) and width (**b**) of grown micro-pillars as a function of irradiation time for samples (made of 97 vol.% CP-ester, 3 vol.% crosslinker) swollen with a monomer solution (97 vol.% CP-ester, 3 vol.% crosslinker, 20 mg/mL cis-Ru-1), under irradiation of circular light beam of varying diameters. Optical microscopic images of the side views of the pillars grown under a light beam with 50 µm (**c**) diameter and 1000 µm diameter (**d**).

Simulation of local growth: Figure S13a shows that the pillar grown under an irradiation diameter of 0.05 mm has a height smaller than the heights generated under bigger irradiation sizes (note that the depth of light penetration into the sample is set the same for all, 100 µm, based on experimental observation). This agrees well with the result in **Figure S12**. Additionally, pillars grown under smaller irradiation sizes (0.05 mm and 0.5 mm) have a height at the center bigger than the edges, whereas the pillar grown under a larger size (2 mm) has a slightly taller edge than the center. Such irradiation size's influence on pillar geometry is because the edges have a monomer supply from both beneath and the sides, whereas the center only has a monomer supply from underneath. The influence of diffusivity of the monomer solution is hard to investigate experimentally, especially considering monomer diffusivity also directly influences the polymerization kinetics of ROMP, but it can be readily investigated with our simulation by changing diffusivity while keeping the polymerization kinetics the same. **Figure S13b** shows that with a faster monomer solution diffusivity, the micropillar has a bigger height. Also, the phenomenon of higher height near edges than in the center is more evident for smaller diffusivity.

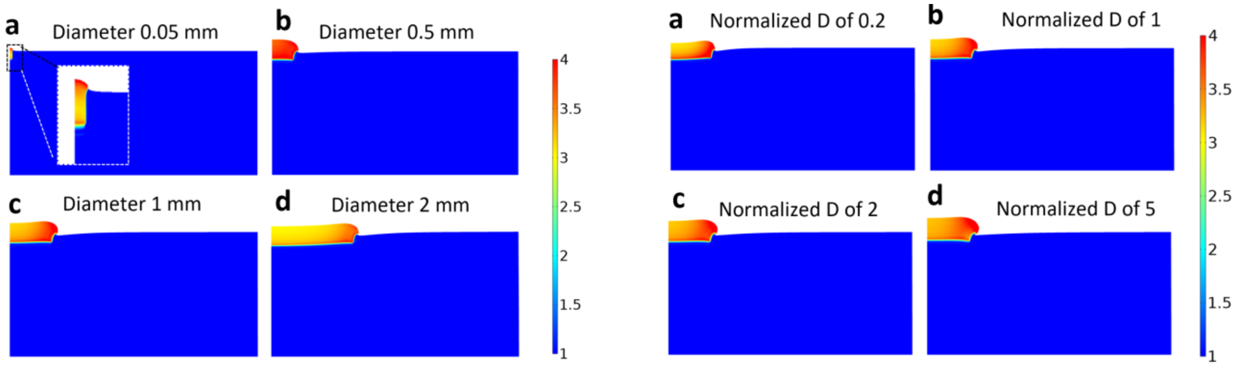

Fig S13. Simulation of local growth: **a** The simulated local growth with irradiation diameter of (i) 0.05 mm, (ii) 0.5 mm, (iii) 1 mm, and (iv) 2 mm. **b** The simulated local growth with normalized monomer solution diffusivity D of (i) 0.2, (ii) 1, (iii) 2, and (iv) 5. The legend bar indicates the dry mass change.

Width change over multiple generations of growth without additional supply of monomer solution: Fig S14 shows the width of the pillars grows slightly larger over more generations of growth, which is because the light-activated catalyst in the pillar stays active and polymerizes the swollen monomer solution outside the light-irradiated region. Overall, the width is not significantly different from the 500 μm diameter of a light dot.

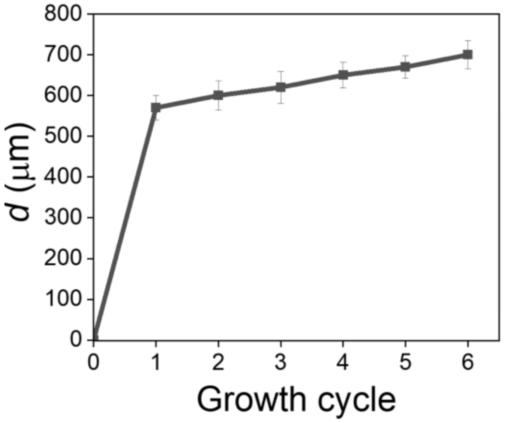

Fig S14. The half-height width *d* of grown pillars over multiple times of growth.

To introduce a specific amount of cis-Ru-1 into the poly(CP-ester) (made of 98 vol.% CP-ester, 2 vol.% crosslinker) for light-induced degradation, we first quantify the swelling ratio of poly(CP-ester) in dichloromethane (DCM) (**Figure S15**). Based on the swelling ratio, a certain amount of cis-Ru-1 was added into DCM and swollen into poly(CP-ester), which gives a certain weight percentage of cis-Ru-1 in the poly(CP-ester) infused with cis-Ru-1 after DCM evaporation.

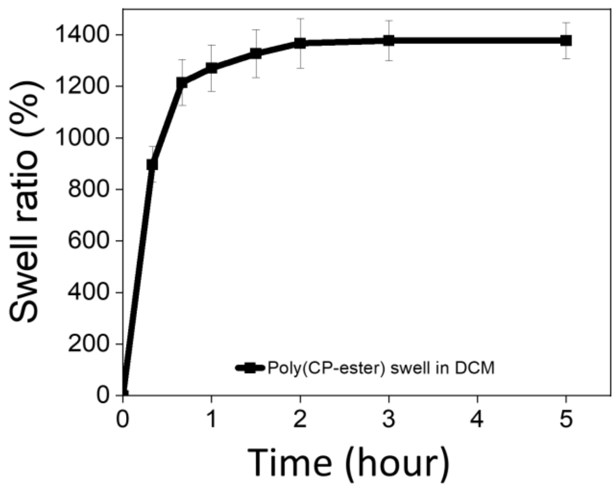

Fig S15. Swelling ratio of poly(CP-ester) with 2 vol.% crosslinker in DCM.

Higher concentration of cis-Ru-1 infilled into poly(CP-ester) would lead to higher concentration of active catalyst under light, and thus faster reactions and deeper depth after the same time of light irradiation (**Figure S16**).

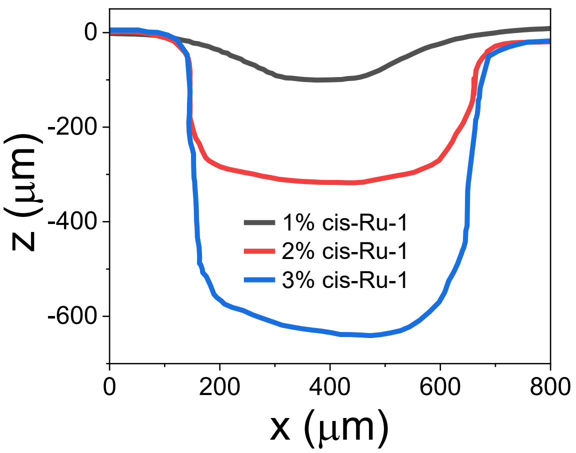

Fig S16. The micro-hole inner surface cross-section profiles on poly(CP-ester) samples (made of 98 vol.% CP-ester, 2 vol.% crosslinker) infused with different amounts of cis-Ru-1 ("1%" means the sample has 1 wt.% of cis-Ru-1 and 99 wt. % of poly(CP-ester)), generated by irradiating light (405 nm, 500 μm diameter, 4800 mW/cm^2) for 6 minutes.

Photo-thermal effect during local degradation: The temperature distribution under light irradiation was quantified using a thermal camera (**Figure S17**). As shown in **Figure S17b**, larger irradiation areas resulted in higher temperatures at the center of the illuminated region. This effect is attributed to both the greater total energy input and reduced cooling efficiency due to slower thermal conduction to the surrounding cooler regions. As shown in **Figure S17c**, increasing the light intensity led to a slight rise in temperature. Additionally, higher catalyst concentrations produced elevated temperatures (**Figure S17d**), likely due to the efficient light absorption and photothermal properties of the catalyst.

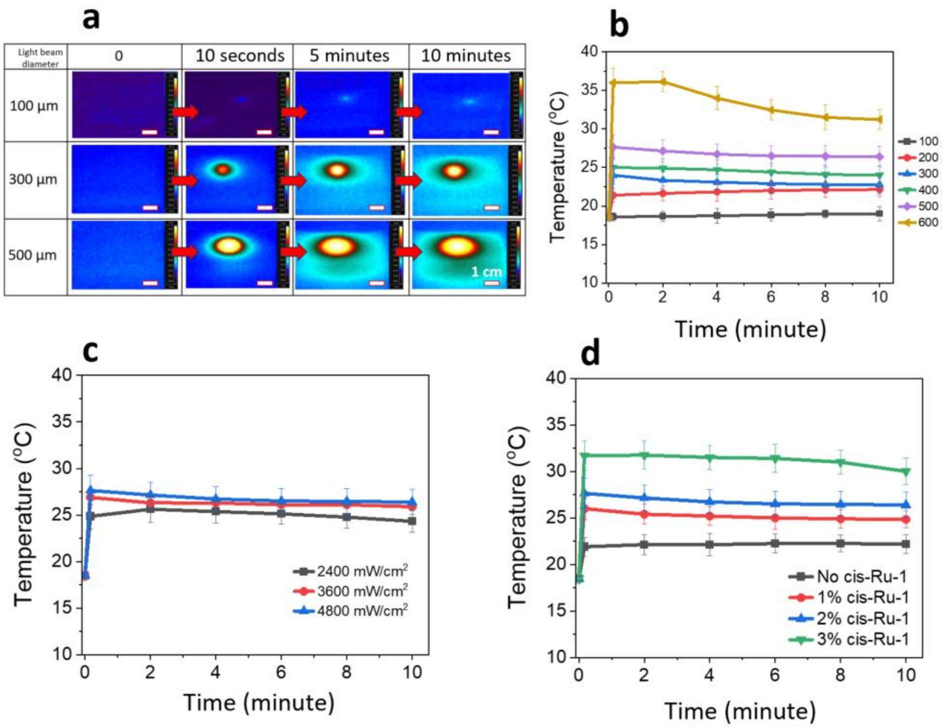

Fig S17. Photo-thermal effect during degradation. **a** The thermal camera images showing the local temperature change on samples under light (with varying light beam diameter, 4800 mW/cm^2) irradiation over time, all samples with 2 wt.% cis-Ru-1. **b** The change of the temperature (the highest temperature at the center of the light irradiated region) over irradiation time for different circular light beam diameters (samples with 2 wt.% cis-Ru-1). **c** The temperature change under different light intensities (500 µm diameter, sample with 2 wt.% cis-Ru-1). **d** The temperature change for samples with different amount of cis-Rui-1 (500 µm diameter, 4800 mW/cm^2), where in the label of "1%" means the sample has 1 wt.% of cis-Ru-1 and 99 wt. % of poly(CP-ester).

Thermal activation of cis-Ru-1: The resolution of light-induced degradation could be reduced if the photocatalyst can be initiated by photo-thermal effect and when the heat is conducted to the non-irradiated region. Thus, we measured the effectiveness of cis-Ru-1 initiating polymerization of CP-ester at different temperatures purely by heat, without light irradiation. **Figure S18** shows that cis-Ru-1 cannot be effectively initiated to polymerize CP-ester even at 80 °C for 10 minutes. This further explains the close match between light irradiation size and micro-hole size during photo-degradation.

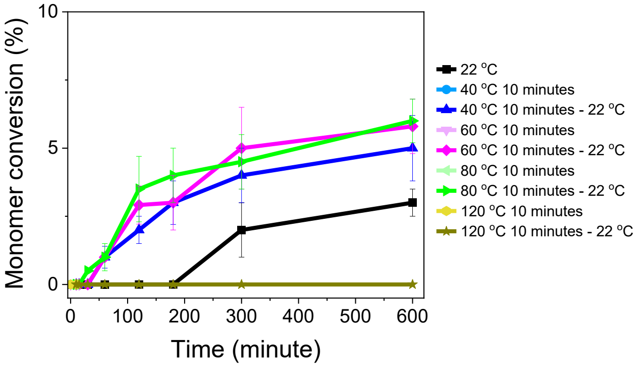

Fig S18. The CP-ester monomer conversion over time upon reacting with cis-Ru-1 (10 mg/mL) at 22 °C (noted as "22 °C"), or after heating to different temperatures for 10 minutes (noted as "x °C for 10 minutes"), followed by sitting at 22 °C for different time (noted as "x °C for 10 minutes - 22 °C ").

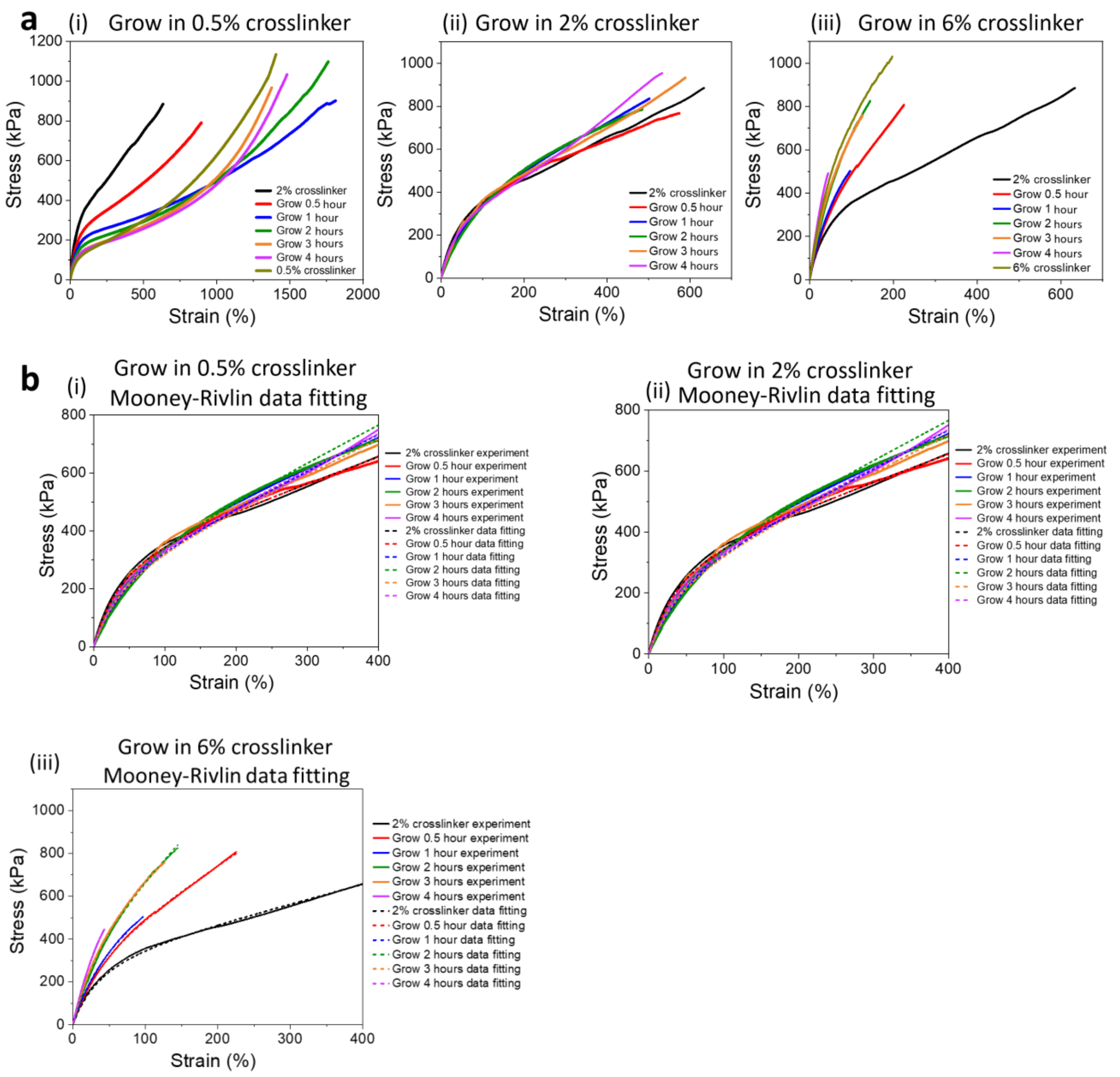

Fig S19. Growth induced mechanical property change. a The tensile testing results of poly(CP-ester) with 2 vol.% crosslinker (named as "2% crosslinker") and after poly(CP-ester) growing in a solution with (i) 0.5 vol.% crosslinker, (ii) 2 vol.% crosslinker, and (iii) 6 vol.% crosslinker for a certain duration. **b** The data fitting of the tensile properties for poly(CP-ester) grown in solutions with (i) 0.5 vol.% crosslinker, (ii) 2 vol.% crosslinker, and (iii) 6 vol.% crosslinker based on a Mooney-Rivlin model.

Degrowth performance: The changes in mechanical properties during degrowth for samples with varying crosslinker concentrations are discussed in the main text. As shown in **Figure S20c**, samples with higher crosslinker density exhibit a slower rate of mass loss and a higher remaining mass. The reduced mass loss rate is likely due to slower depolymerization in the more highly crosslinked and mechanically rigid networks, which can hinder molecular mobility and thereby limit cross-metathesis depolymerization reactions. The increased remaining mass is attributed to

the presence of non-volatile crosslinkers, which do not evaporate like the monomer species and thus persist in the material, as demonstrated in **Figure S6**.

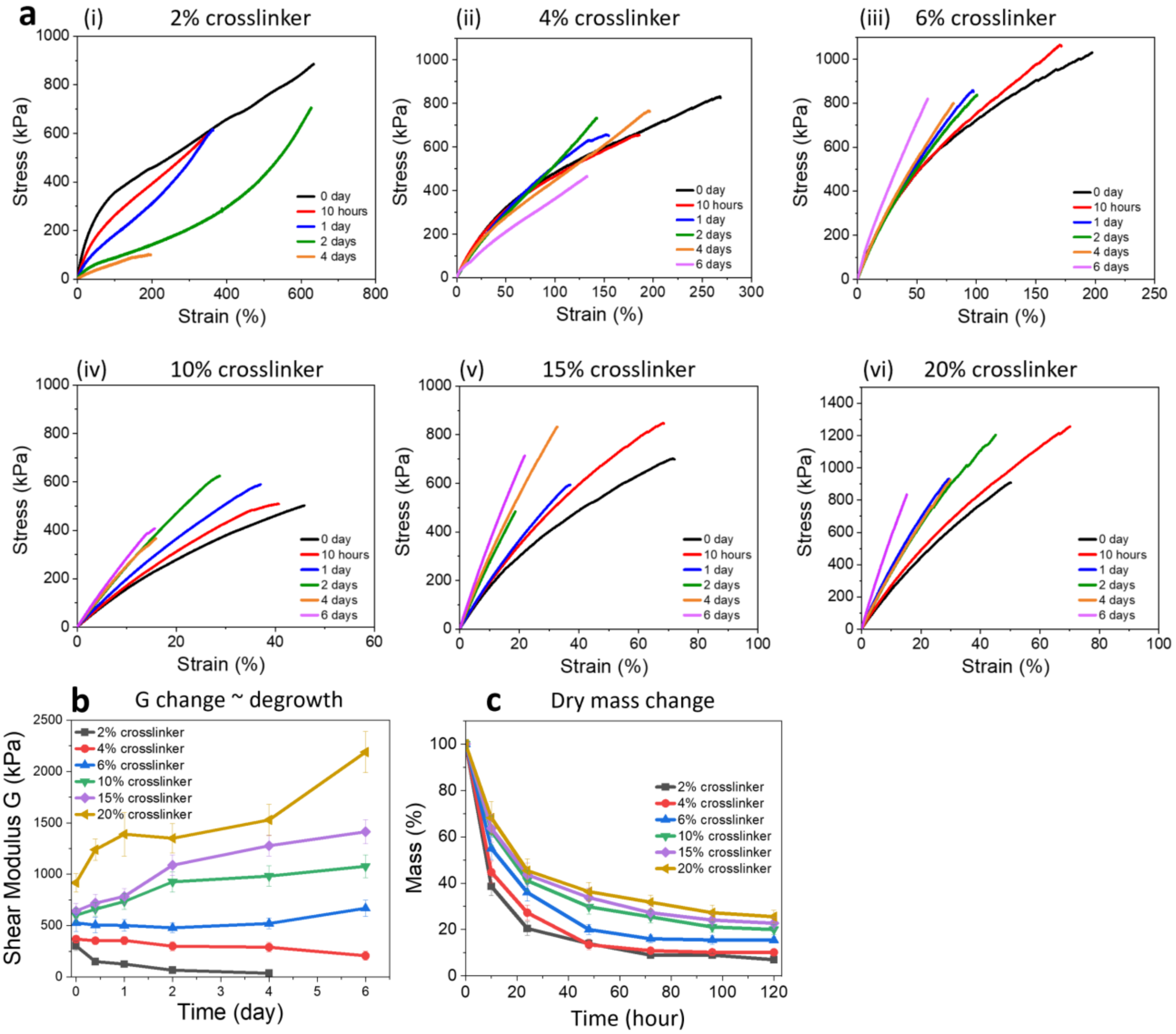

Fig S20. Degrowth-induced tuning of mechanical properties. **a** The tensile stress-strain behavior change over degrowth in the setup of **Figure S6a** for a certain time for poly(CP-ester) with crosslinker density of (i) 2 vol.%, (ii) 4 vol.%, (iii) 6 vol.%, (iv) 10 vol.%, (v) 15 vol.%, and (vi) 20 vol.%. **b** The shear modulus change upon degrowth. **c** The mass change upon degrowth. The mass change is based on dry polymer weight after washing samples at each state in 90 vol.% DCM and 10 vol.% EVE, and drying for 24 hours.

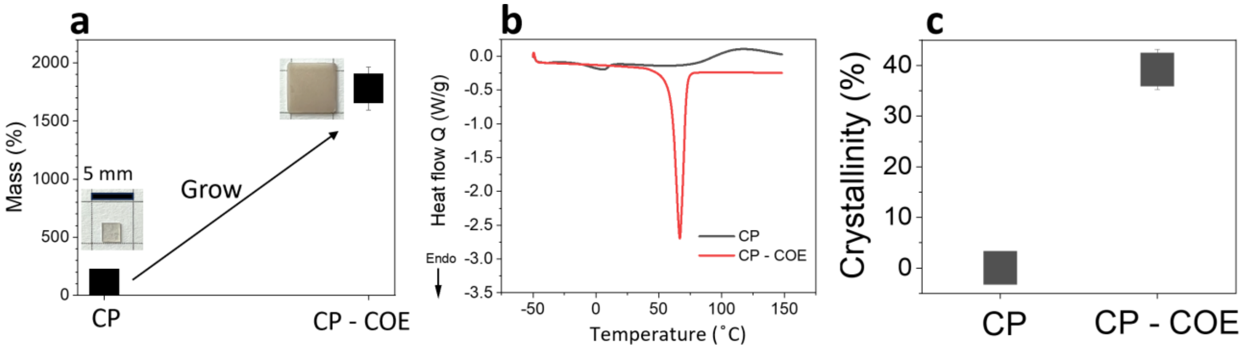

Fig S21. Grow in COE. **a** The mass change of poly(cyclopentene) (noted as "CP")) before and after growth in cyclooctene (COE) (noted as "CP-COE"). **b** The DSC spectra of CP and CP-COE. **c** The crystallinity of CP and CP-COE.

Degrowth performance of copolymers of COE and CP-ester

Thin films (1 mm thick) of copolymers composed of COE, CP-ester, 2 vol.% crosslinker, and 10 mg/mL G2 were subjected to degrowth immediately following 3 hours of polymerization, using the setup shown in **Figure S7a**. At predetermined time points, a portion of each film was excised and immersed in a solution of 90 vol.% DCM and 10 vol.% EVE to deactivate G2, followed by washing and drying for 24 hours prior to tensile testing. As shown in **Figure S22a**, the shear modulus of copolymers containing varying COE contents (10–70 vol.%) all increases over time during degrowth. Samples with higher COE content exhibited greater final shear modulus values. This increase is attributed to the depolymerization of poly(CP-ester) into CP-ester monomer and its subsequent evaporation, which enriches the remaining material in poly(COE). As poly(COE) becomes the dominant component, it can crystallize more effectively, thereby stiffening the material (**Figure S22c**). Initially, the shear modulus values for samples with 10%, 30%, and 70% COE were relatively similar—0.2 MPa, 0.5 MPa, and 0.97 MPa, respectively—and all below 1 MPa. These comparable initial values suggest random copolymerization of COE and CP-ester, which prevents effective poly(COE) crystallization at the outset. However, significant differences emerged during degrowth. After 5 days, the shear modulus of the 10% COE sample increased from 0.2 MPa to 1.2 MPa, while the 70% COE sample increased dramatically from 0.97 MPa to 40 MPa (**Figure S22a(v)**). This disparity is likely due to differences in the poly(COE) block length within the copolymer chains. Higher initial COE content yields longer poly(COE) segments that can more readily crystallize once the poly(CP-ester) blocks are removed. In contrast, lower COE content results in shorter poly(COE) segments, which are less likely to associate and form crystalline domains, thus limiting mechanical enhancement during degrowth. This combination of initially similar mechanical properties and divergent behavior upon degrowth offers a promising strategy for designing heterogeneous materials. Such materials can be fabricated with a uniform mechanical profile initially, but develop spatially varied properties over time, enabling applications that benefit from controlled property evolution.

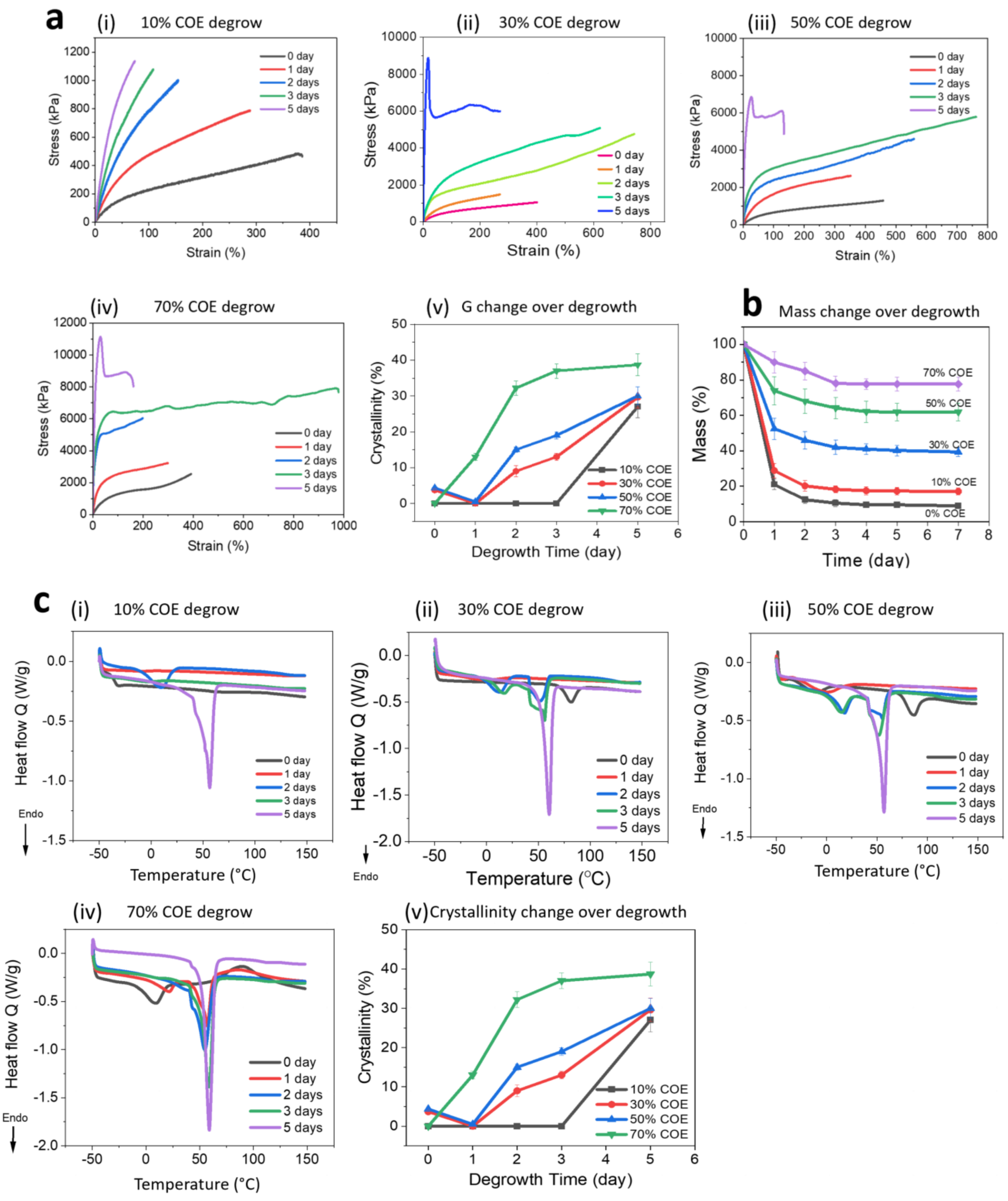

Figure S22. Degrowth of copolymers of COE and CP-ester. **a** Tensile property change along degrowth for a certain time for copolymers made of (i) 10 vol.% COE, 88 vol.% CP-ester, 2 vol.% crosslinker (noted as “10% COE”), (ii) 30 vol.% COE, 68 vol.% CP-ester, 2 vol.% crosslinker (noted as “30% COE”), (iii) 50 vol.% COE, 48 vol.% CP-ester, 2 vol.% crosslinker (noted as “50% COE”), (iv) 70 vol.% COE, 28 vol.% CP-ester, 2 vol.% crosslinker (noted as “70% COE”). (v) Shear modulus G change after degrowth for a certain time. **b** Dry mass change after degrowth for a certain time. **c** DSC spectra along the degrowth process for samples of (i) 10% COE, (ii) 30% COE, (iii) 50% COE, (iv) 70% COE. (v) Crystallinity change along the degrowth process for samples with varying amounts of COE.

Repeated degrowth and regrowth

The modulation of mechanical properties in the CP-ester/COE copolymer system is not strictly reversible, as it arises from a dynamic polymer network in which growth and degrowth involve continuous bond breaking and reformation accompanied by network restructuring, and the polymerization rate of COE is significantly faster than CP-ester. Additionally, regrowth requires elevated temperatures (i.e. 60 °C) to disrupt existing crystalline domains, enabling network expansion and additional monomer uptake. To examine repeated growth–degrowth behaviors and the resulting mechanical property changes, two regrowth scenarios were investigated: regrowth in pure CP-ester monomer (**Figure S23a**) and regrowth in a CP-ester/COE mixture (**Figure S23b**). When regrown in pure CP-ester and upon a second degrowth, the material fails to recover the high stiffness observed in the first degrowth cycle (**Figure S23 a(ii)**). This is because extensive chain exchange between poly(COE) and poly(CP-ester) happens during the regrowth and the subsequent degrowth process, which restrict efficient reformation of the crystalline domains of poly(COE) (**Figure S23 a(iv)**). The high crystallinity realized after the first degrowth (**Figure S23 a(iv)**) is because that COE has much higher polymerization rate than CP-ester during the initial polymerization/sample preparation process, forming long blocks of poly(COE) chains that efficiently recrystallized after extraction of poly(CP-ester) upon degrowth. On the other hand, regrowth in the CP-ester/COE mixture enables newly absorbed COE to form new crystalline domains during the second degrowth, restoring a high modulus comparable to that of the first cycle. It should be noted, however, that in this case the regrowth extent must significantly exceed the original sample mass to compensate for the permanently disrupted crystalline domains formed during the first regrowth.

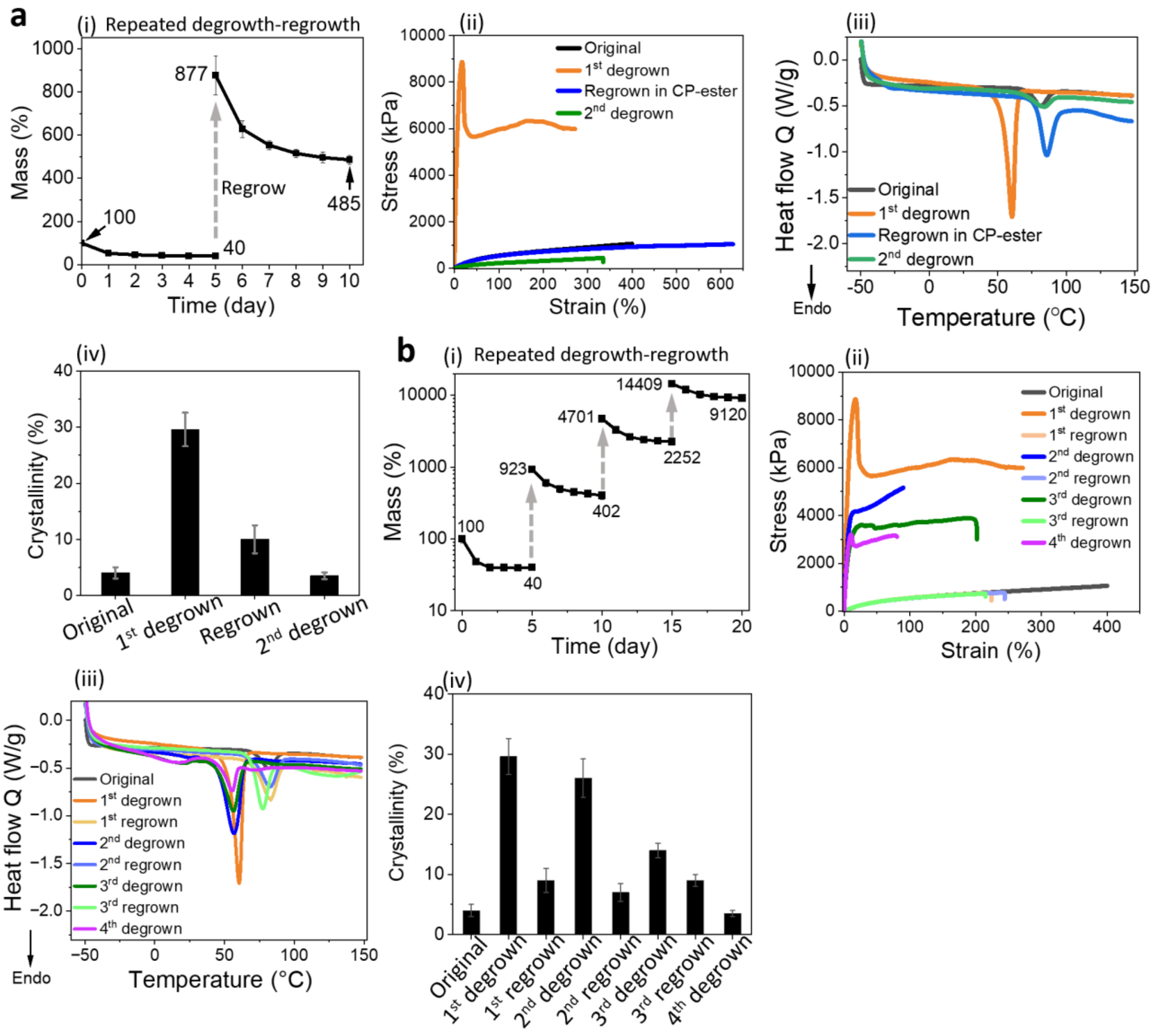

Figure S23. Repeated degrowth and regrowth. **a** The (i) mass change, (ii) tensile properties, (iii) DSC spectra and (iv) crystallinity change over degrowth of "30% COE" (denoted as "Original" in **Figure S23a**, made of 68 vol.% CP-ester, 2 vol.% Crosslinker, 30 vol.% COE, 10 mg/mL G2) for 5 days (denoted as "1st degrown") with the setup of **Figure S7a.** The properties after regrowth in a CP-ester solution (98 vol. CP-ester, 2 vol.% Crosslinker, G2), denoted as "Regrown in CP-ester" or "Regrown". The properties after a second degrowth for 5 days (denoted as "2nd degrown"). **b** The (i) mass change, (ii) tensile properties, (iii) DSC spectra and (iv) crystallinity change over the repeated degrowth of "30% COE" (denoted as "Original") and regrowth in a CP-ester/COE mixture (68 vol.% CP-ester, 30 vol.% COE, 2 vol.% Crosslinker, G2). The numbers in **Figure S23 a(i), b(i)** indicate the mass in the beginning and after 5 days of degrowth of each cycle. More details for regrowth in the CP-ester solution are shown in the Experimental Section in main text. The regrowth in the CP-ester/COE mixture follows the same procedure as regrowth in CP-ester solution, differing only in the solution (68 vol.% CP-ester, 30 vol.% COE, 2 vol.% Crosslinker, 13 mg/mL G2) used for swelling at 4 °C. Error bars represent the standard deviation from 3 measurements.

The stability of the dry polymers over storage in air

The polymers made of ROMP have double bonds on chains that are susceptible to oxidation, which will decrease the mechanical stability. The mechanical stability can be improved through technologies like adding antioxidants[6] and modifying monomers with antioxidants,[7] which is not the scope of this work. Here, we report the mechanical stability of poly(CP-ester) without adding antioxidants (**Figure S24**).

Figure S24a(i) shows that 2% crosslinker sample have similar mechanical properties in the first 30 days of storage on bench in open air, and then the samples become stiffer and show shorter breaking strain. **Figure S24a(ii-v**) shows that the copolymers of COE and CP-ester exhibit a trend of decreasing breaking strain, and a gradual increase in Young's modulus over a longer time of storage. Similar trends of decreasing breaking strain and increasing Young's modulus over oxidative reactions are also observed for ROMP-based poly(dicyclopentadiene).[8] These changes can be attributed to the oxidative reactions of the double bonds on polymer chains, some of which can cause increased crosslinking between polymer chains that embrittle the polymer[8]. The oxidation is verified with Fourier transform infrared (FTIR) spectroscopy shown in **Figure S24b**. Particularly, the broad absorption peak centered around 3400 cm^{-1} represents various double bond-oxidation products[8] such as alcohols, carboxylic acids and hydroperoxides, etc. Interestingly, the 90% COE (90 vol.% COE, 8 vol.% CP-ester, 2 vol.% crosslinker) sample shows barely noticeable change in Young's modulus after 60 days. The oxidation peaks centered around 3400 cm^{-1} also do not show up, indicating minimal oxidation, which could be attributed to the much slower O_2 permeation through the poly(COE) sample with a high level of crystallization.

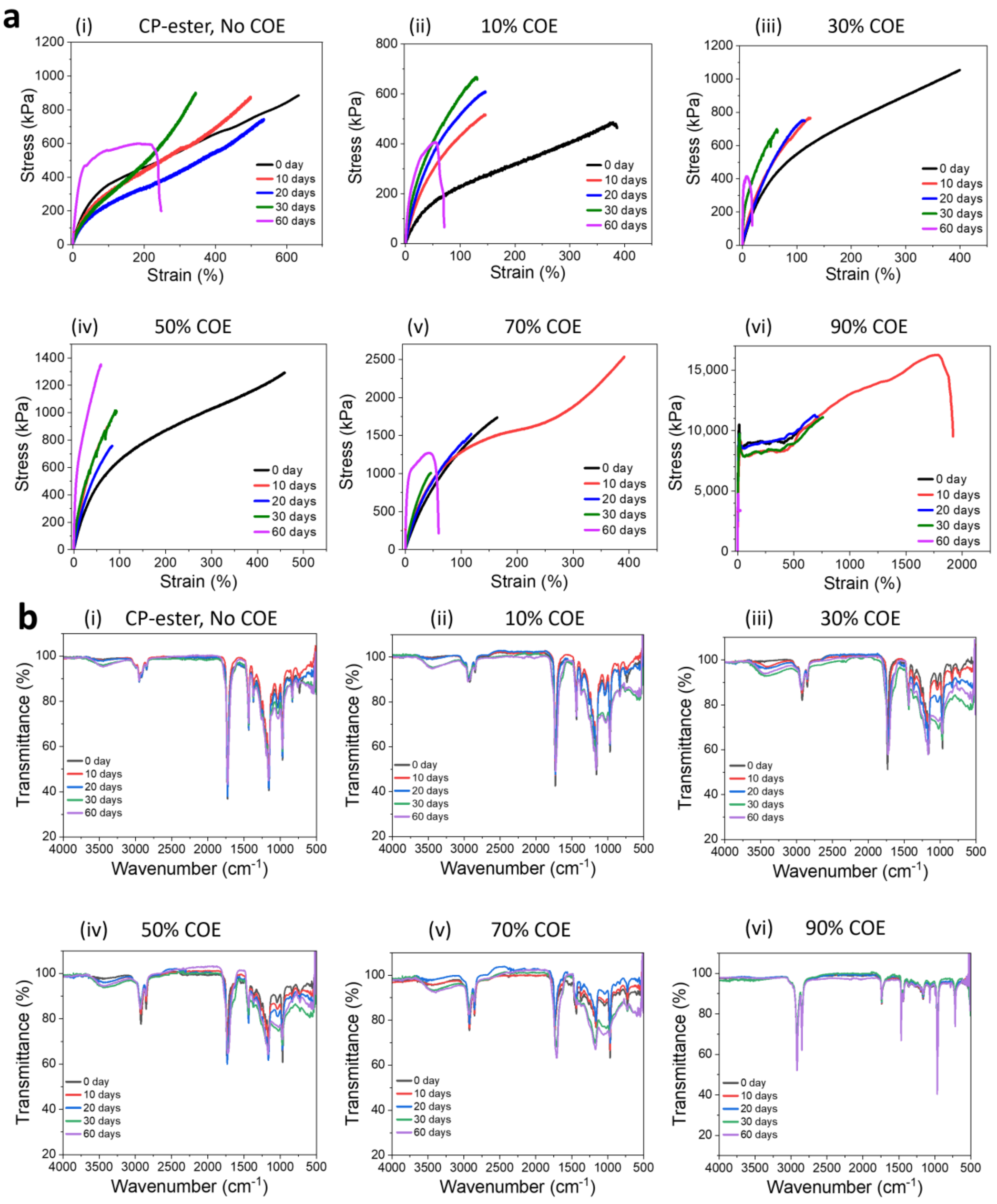

Figure S24. Mechanical properties of the polymers over storing in air. a (i) Tensile stress-strain behaviors of poly(CP-ester) with 2 vol.% crosslinker sitting on bench in open air for a certain time. Tensile properties of copolymers made of (ii) 10 vol.% COE, 88 vol.% CP-ester, and 2 vol.% crosslinker (noted as "10% COE"), (iii) 30 vol.% COE, 68 vol.% CP-ester, and 2 vol.% crosslinker (noted as "30% COE"), (iv) 50 vol.% COE, 48 vol.% CP-ester, and 2 vol.% crosslinker (noted as 50% COE), (v) 70 vol.% COE, 28 vol.% CP-ester, and 2 vol.% crosslinker (noted as "70% COE"), (vi) 90 vol.% COE, 8 vol.% CP-ester, and 2 vol.% crosslinker (noted as "90% COE") sitting on bench for a certain time. **b** (i) FTIR spectra of poly(CP-ester) with 2 vol.% crosslinker sitting in the air for a certain time. FTIR spectra of copolymers (ii) 10% COE, (iii) 30 COE, (iv) 50% COE, (v) 70% COE, (vi) 90% COE sitting on bench for a certain time.

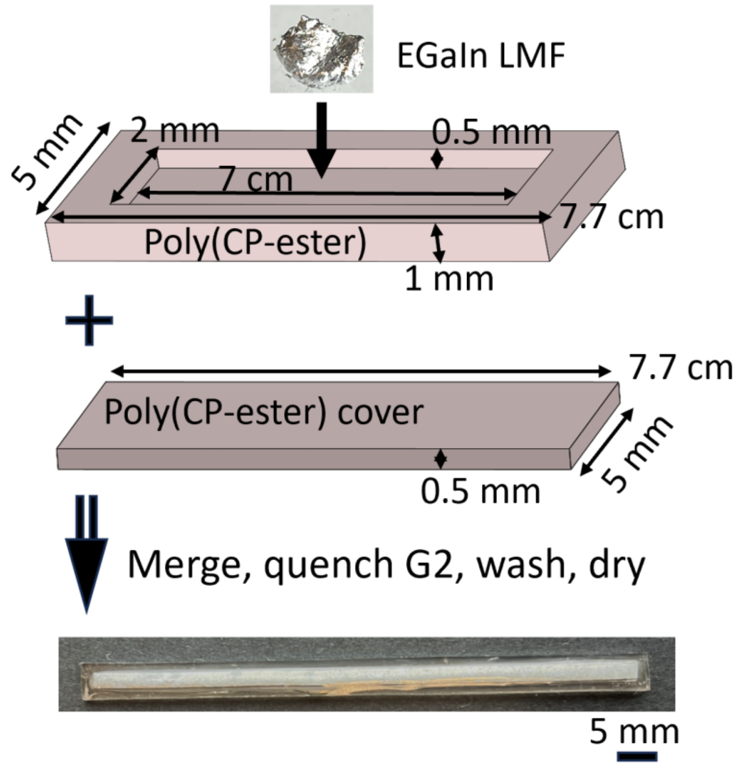

Figure S25. Process of making an antenna through embedding EGaIn LMF into the channel inside poly(CP-ester). The two poly(CP-ester) films were first polymerized for 20 minutes, followed by infilling EGaIn LMF, and then merged together by 2 hours more of polymerization and interfacial chain exchange.

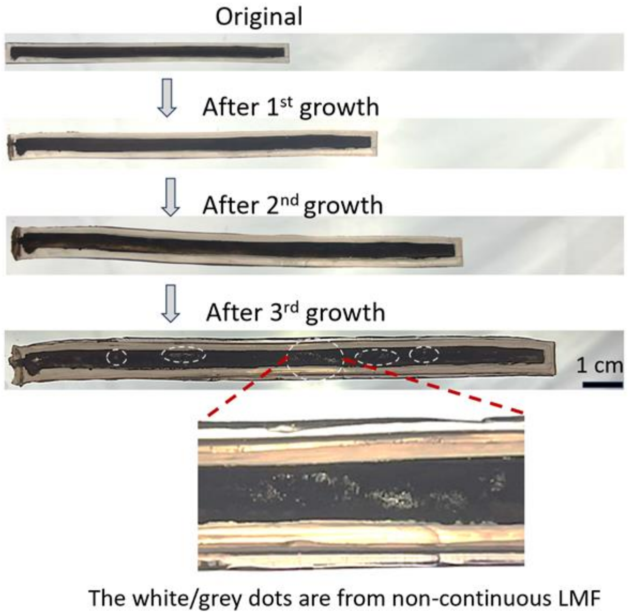

Figure S26. The images of the antenna in the original state and after a few times of growth (all wash, dried samples), showing the change of continuity of EGaIn LMF. The images were acquired by passing light through the samples and onto the camera sensor.

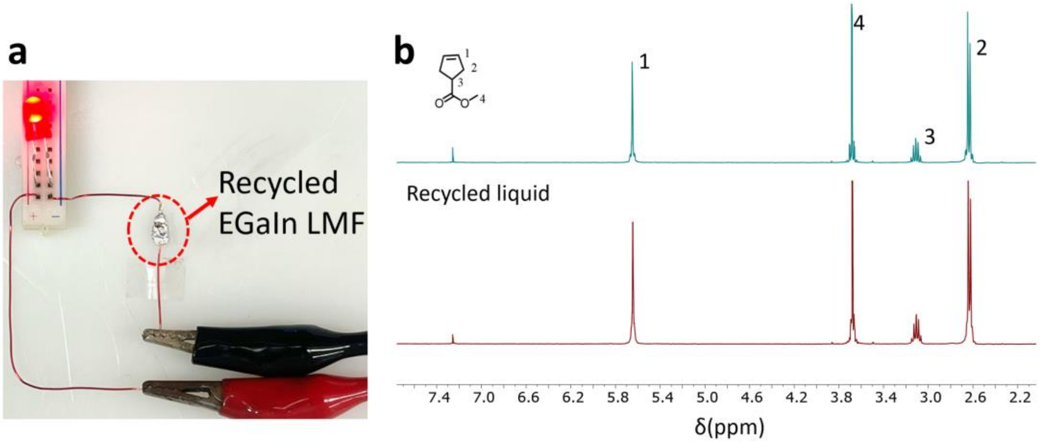

Figure S27. a The EGaIn LMF from recycling antenna has good conductivity to connect the circuit to light up the LED light. **b** The ^{1}H-NMR spectra of recycled CP-ester and purchased CP-ester.

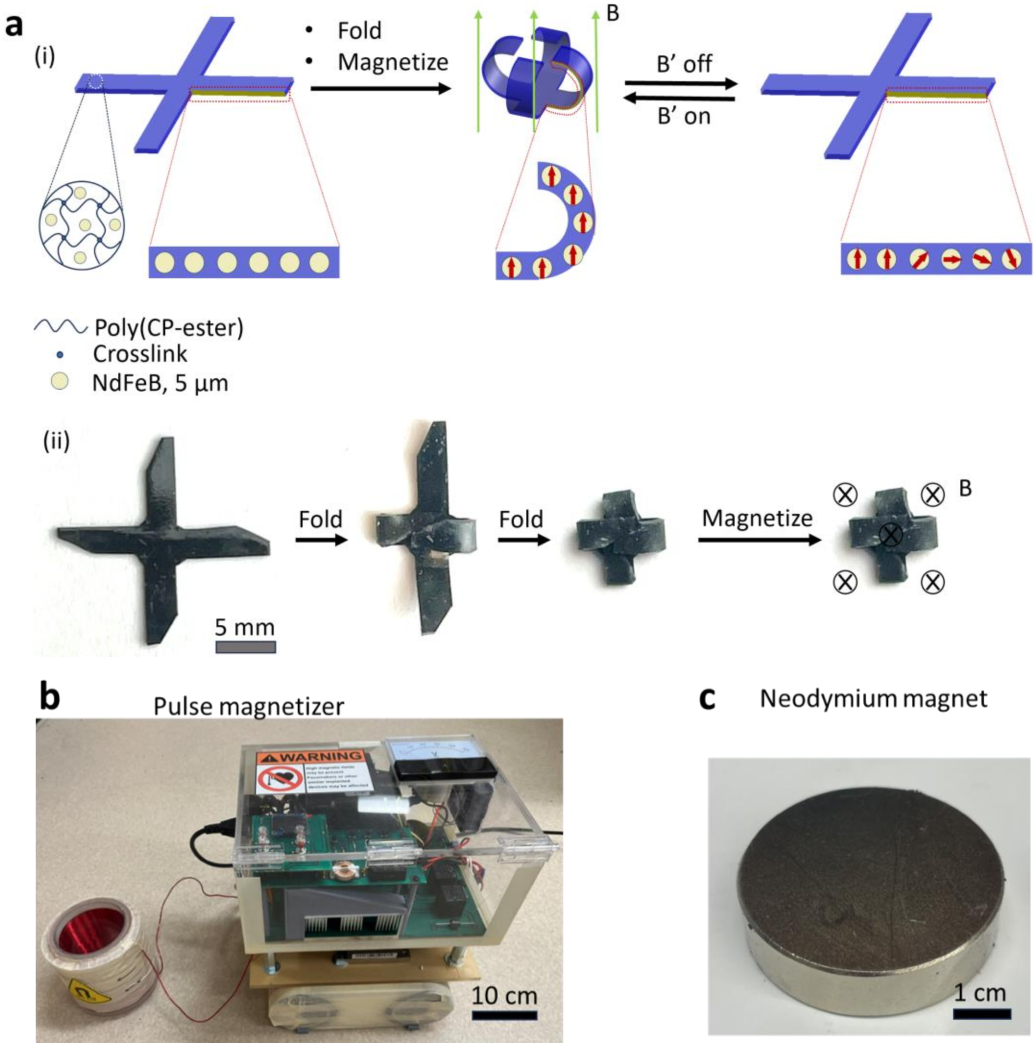

Figure S28. a (i) Schematic and (ii) experimental results showing the folding and magnetization of the magnetic soft robot to design the remaining magnetic dipole distribution. **b** Lab-made pulse magnetizer (~1.5 T at the electromagnetic coil center). **c** The permanent magnet used to actuate the magnetized soft robot from underneath the table.

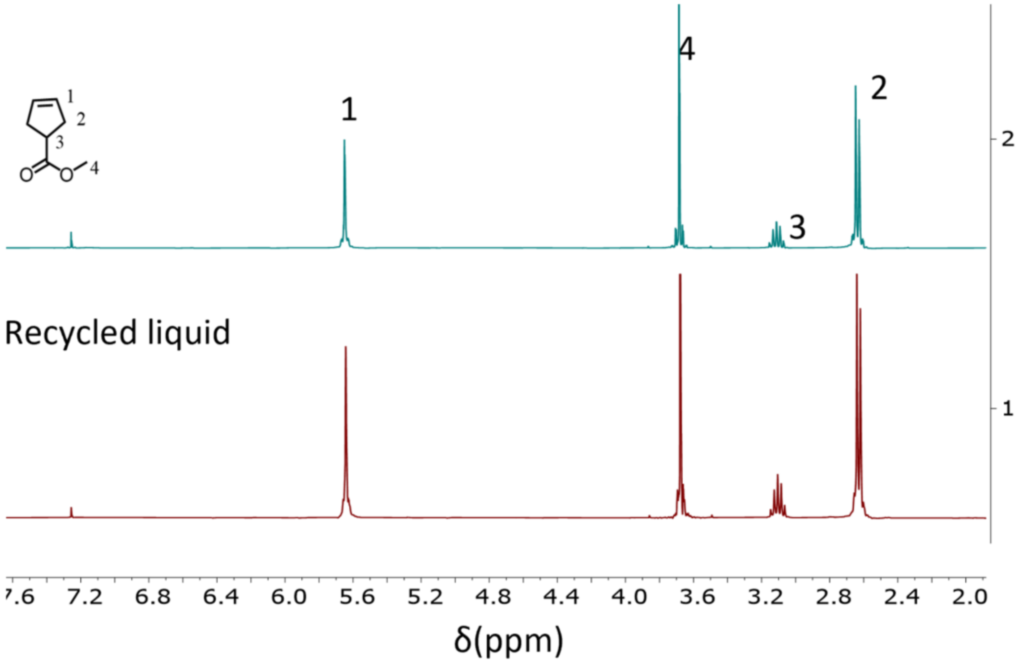

Figure S29. The ^{1}H-NMR spectra of recycled CP-ester and pure CP-ester.

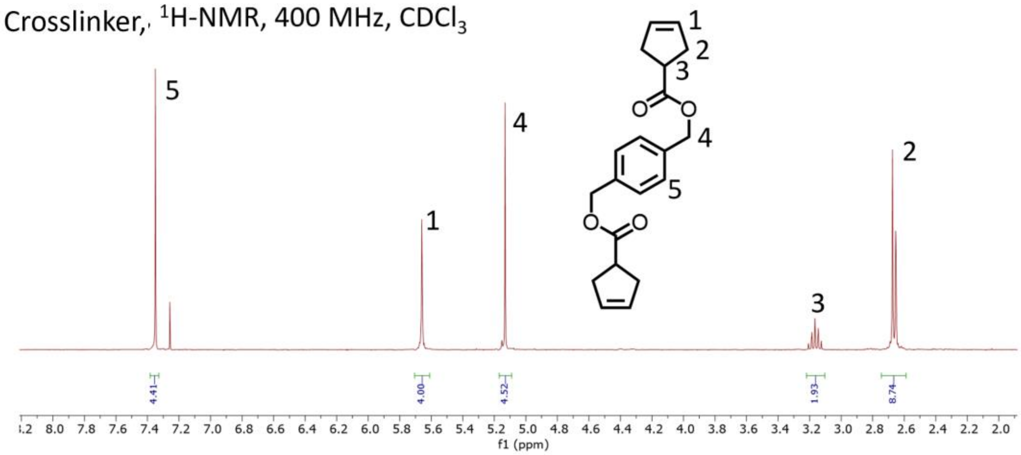

Figure S30. ^{1}H-NMR spectrum of the synthesized crosslinker in $CDCl_3$.

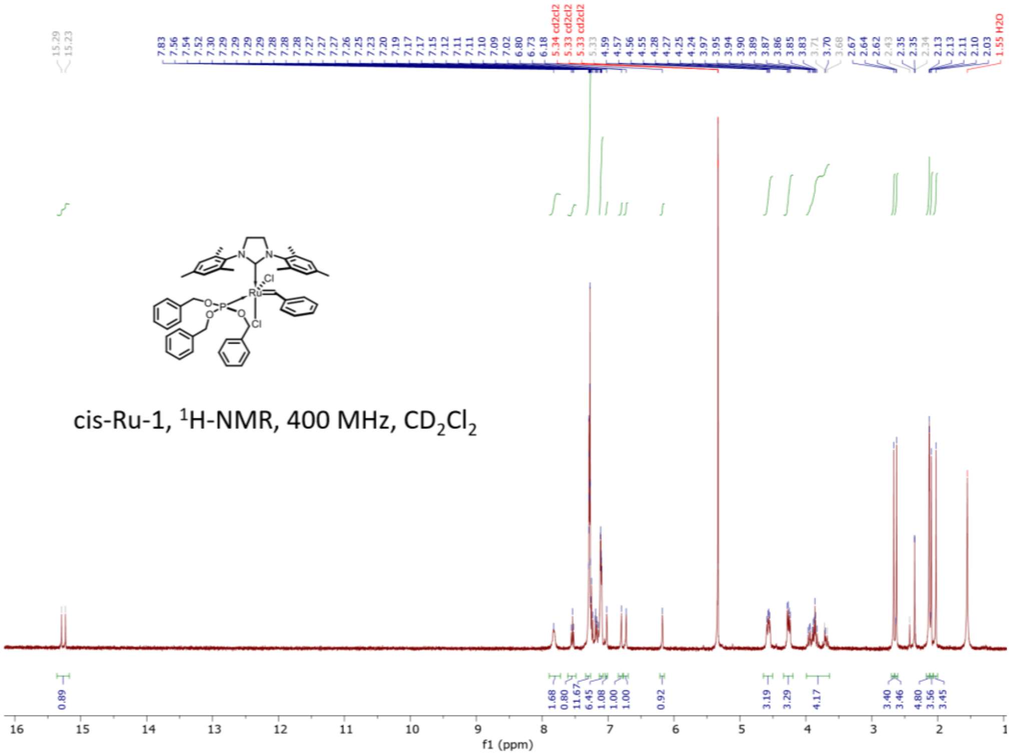

Figure S31. ^{1}H-NMR spectrum of the synthesized cis-Ru-1 in CD_2Cl_2.

Video S1. Growable soft magnetic robot.

References

[1] Liu, H. et al. Dynamic remodeling of covalent networks via ring-opening metathesis polymerization. ACS Macro Letters 7, 933-937 (2018).

[2] Eivgi, O., Vaisman, A., Nechmad, N. B., Baranov, M. & Lemcoff, N. G. Latent Ruthenium Benzylidene Phosphite Complexes for Visible-Light-Induced Olefin Metathesis. ACS Catalysis 10, 2033-2038 (2019).

[3] Sanford, M. S., Love, J. A. & Grubbs, R. H. A versatile precursor for the synthesis of new ruthenium olefin metathesis catalysts. Organometallics 20, 5314-5318 (2001).

[4] Zhang, H. & Hu, Y. A statistical-chain-based theory for dynamic living polymeric gels with concurrent diffusion, chain remodeling reactions and deformation. Journal of the Mechanics and Physics of Solids 172, 105155 (2023).

[5] Leguizamon, S. C. et al. Photoinitiated olefin metathesis and stereolithographic printing of polydicyclopentadiene. Macromolecules 55, 8273-8282 (2022).

[6] Nicolas, C. et al. ROMP of novel hindered phenol-functionalized norbornenes and preliminary evaluation as stabilizing agents. Polymer Degradation and Stability 186, 109522 (2021).

[7] Lambeth, R. H. & Baranoski, M. H. Oxidative Stabilization of Poly (norbornene) Polymers Prepared by Ring Opening Metathesis Polymerization. Journal of Macromolecular Science, Part A 51, 962-965 (2014).

[8] Huang, J. et al. Thermal oxidation of Poly (dicyclopentadiene)–kinetic modeling of double bond consumption. Polymer Degradation and Stability 166, 258-271 (2019).
